# The combination of simethicone and N-acetylcysteine in lidocaine ice popsicle compared with lidocaine ice popsicle alone in patients undergoing unsedated esophagogastroduodenoscopy: A prospective double-blind randomized controlled trial

**DOI:** 10.1371/journal.pone.0361495

**Published:** 2026-10-26

**Authors:** Pailin Intarasangkhanawin, Saritphat Orrapin, Worapong Anuponganan, Neranchala Soonthornkes, Prasit Mahawongkajit

**Affiliations:** 1 Department of Surgery, Faculty of Medicine, Thammasat University, Pathum Thani, Thailand; 2 Department of Anesthesiology, Faculty of Medicine, Thammasat University, Pathum Thani, Thailand; University of Nebraska Medical Center College of Medicine, UNITED STATES OF AMERICA

## Abstract

**Background:**

Optimal mucosal visualization is essential for high-quality esophagogastroduodenoscopy (EGD), particularly for the detection of subtle upper gastrointestinal lesions. Oral simethicone and N-acetylcysteine (NAC) are commonly used to improve mucosal cleanliness, whereas lidocaine ice popsicles provide effective topical pharyngeal anesthesia for unsedated EGD. We evaluated whether incorporating simethicone and NAC into a lidocaine ice popsicle could improve mucosal visibility while maintaining patient comfort.

**Methods:**

This single-center, prospective, double-blind, randomized controlled trial enrolled 374 adults undergoing unsedated diagnostic EGD between December 2024 and June 2025. Participants were randomized (1:1) to receive either a lidocaine ice popsicle containing simethicone (100 mg) and NAC (600 mg) or a conventional lidocaine ice popsicle. The primary outcome was mucosal visibility assessed using a modified Total Visibility Score (TVS). Secondary outcomes included procedure time, water volume required for mucosal cleansing, additional simethicone use, endoscopist satisfaction, ease of esophageal instrumentation, gag reflex, patient satisfaction, discomfort, pain, and adverse events. Statistical analyses were performed using SPSS version 25.0 (IBM Corp., Armonk, NY, USA).

**Results:**

All 374 randomized participants (187 per group) completed the study and were analyzed according to their assigned groups. Baseline characteristics were descriptively similar between groups. The intervention group had a significantly lower TVS than the control group (6.19 ± 0.51 vs. 10.39 ± 2.22; mean difference, −4.20; 95% CI, −4.53 to −3.88; P < 0.001), indicating better mucosal visibility. Exploratory segment-specific analyses showed lower visibility scores in all six gastrointestinal segments. The intervention group also had higher endoscopist satisfaction, shorter procedure time (10.87 ± 1.44 vs. 12.75 ± 0.96 min; P < 0.001), lower water requirements for mucosal cleansing, and less frequent use of additional diluted simethicone during EGD (12.8% vs. 96.8%; P < 0.001). Exploratory analyses showed statistically significant differences in gag reflex and pain scores, although the absolute differences were small. Patient satisfaction, discomfort, and ease of esophageal instrumentation did not differ significantly between groups. No adverse events were observed during the defined observation period.

**Conclusions:**

The combined formulation of simethicone and N-acetylcysteine incorporated into a lidocaine ice popsicle improved mucosal visibility compared with a lidocaine ice popsicle alone during unsedated EGD and was associated with improved procedural efficiency. This integrated formulation may provide a practical approach to pre-endoscopic preparation by combining topical anesthesia with mucosal cleansing. Further multicenter studies are warranted to confirm its effectiveness, generalizability, and safety with longer follow-up.

**Trial registration:**

Thai Clinical Trials Registry TCTR20240606004

## Introduction

Upper gastrointestinal endoscopy (esophagogastroduodenoscopy; EGD) plays a pivotal role in the diagnosis of both benign and malignant upper gastrointestinal (GI) diseases. High-quality mucosal visualization during EGD is essential for detecting subtle lesions, including early esophageal and gastric neoplasia, where early diagnosis substantially improves treatment outcomes and patient prognosis [1–6].

Patients undergoing EGD are routinely instructed to fast for at least 6–8 hours before the procedure to minimize gastric contents and reduce aspiration risk [7]. However, despite adequate fasting, residual mucus, foam, bubbles, and gastric secretions frequently remain adherent to the mucosal surface, impairing visualization and potentially obscuring subtle abnormalities. Several pre-endoscopic agents have therefore been investigated to improve mucosal cleanliness, including simethicone as a defoaming agent, mucolytic agents such as N-acetylcysteine (NAC) and pronase, proteolytic enzyme preparations, sodium bicarbonate, and peppermint oil [8–14]. Among these, simethicone and NAC are widely used because of their complementary mechanisms of action in reducing bubbles and dissolving mucus.

In many countries, sedation is increasingly used to improve patient comfort during EGD. Nevertheless, unsedated EGD remains common in many healthcare systems because it is less resource-intensive, allows faster patient turnover, avoids sedation-related adverse events, and reduces healthcare costs [15–17]. Patient anxiety and discomfort may influence tolerance of unsedated EGD, making adequate topical pharyngeal anesthesia an important component of procedural success [18]. Lidocaine is the most commonly used topical anesthetic and is available in several formulations, including spray, viscous solution, lozenges, lollipops, nebulized preparations, and ice popsicles [19–24].

Although oral simethicone solution is commonly administered before EGD to improve mucosal visibility, its effectiveness may be limited by its relatively short contact time with the upper gastrointestinal mucosa. In addition, patient acceptance may be reduced by its unpleasant taste and the need to ingest additional liquid immediately before the examination. A previous study demonstrated that a lidocaine ice popsicle provides prolonged topical anesthetic contact through slow dissolution and a cooling effect, resulting in effective pharyngeal anesthesia for unsedated EGD [24]. Building on this concept, incorporating simethicone and NAC into a slowly dissolving lidocaine ice popsicle may prolong contact of the active agents with the upper gastrointestinal mucosa, combine topical anesthesia with mucosal cleansing, and simplify pre-endoscopic preparation while potentially improving patient acceptance. However, the clinical efficacy of this integrated formulation has not yet been evaluated.

Therefore, this prospective, double-blind, randomized controlled trial aimed to compare a lidocaine ice popsicle containing simethicone and N-acetylcysteine with a conventional lidocaine ice popsicle in patients undergoing unsedated EGD. We hypothesized that the combined formulation would improve gastric mucosal visibility while maintaining patient comfort and procedural tolerability.

## Materials and methods

### Study design and ethical approval

This single-center, prospective, double-blind, randomized controlled trial was conducted at Thammasat University Hospital, Thailand. The study protocol was prospectively registered with the Thai Clinical Trials Registry (TCTR20240606004; registration date: 2 June 2024). Ethical approval was obtained from the Human Ethics Committee of the Faculty of Medicine, Thammasat University (MTU-EC-SU-1–250/65). The study was conducted in accordance with the Declaration of Helsinki, and written informed consent was obtained from all participants before enrollment.

### Participants

Between December 2024 and June 2025, consecutive adult patients scheduled to undergo unsedated diagnostic EGD were prospectively recruited at Thammasat University Hospital.

Eligible participants were aged 20 years or older and underwent EGD for upper gastrointestinal cancer screening or evaluation of upper gastrointestinal symptoms, including dysphagia, dyspepsia, gastroesophageal reflux disease, abdominal pain, and anemia.

Patients were excluded if they had a history of upper gastrointestinal surgery, severe dysphagia, previous corrosive ingestion, pregnancy, psychiatric illness, neuromuscular disorders, unstable vital signs, or known allergy or contraindication to lidocaine, simethicone, or NAC.

### Randomization and blinding

The random allocation sequence was computer-generated by a research assistant who was not involved in participant enrollment or outcome assessment. The research team screened eligible participants and obtained written informed consent before enrollment. Participants were then randomly assigned in a 1:1 ratio to the intervention or control group. Allocation concealment was maintained using sequentially numbered, opaque, sealed envelopes (SNOSE), which were prepared in advance by the research assistant according to the computer-generated allocation sequence. Each envelope contained the group assignment and remained sealed until enrollment was completed. No blocking or stratification was used. After enrollment, the first endoscopy nurse opened the next sequentially numbered envelope and prepared and administered the assigned ice popsicle. This nurse was not involved in outcome assessment.

The intervention and control preparations were produced using identical molds, resulting in identical appearance, size, and packaging. Both formulations were stored under identical conditions and administered using the same protocol. Participants, the performing endoscopist, the assisting endoscopy nurse, investigators responsible for data collection, and the statistician remained blinded to treatment allocation until completion of the final statistical analysis.

### Study intervention

Participants allocated to the intervention group received an ice popsicle prepared from 6 mL of lidocaine viscous solution (20 mg/mL; total lidocaine dose 120 mg), simethicone 100 mg, and N-acetylcysteine 600 mg. Sterile water was added to achieve a final volume of 30 mL before freezing overnight in a standardized mold.

Participants allocated to the control group received an identical ice popsicle prepared from 6 mL of lidocaine viscous solution (20 mg/mL; total lidocaine dose 120 mg) diluted with sterile water to a final volume of 30 mL and frozen using the same procedure.

On the day of endoscopy, the assigned ice popsicle was administered by the first endoscopy nurse. Participants were instructed to suck the ice popsicle completely while in the sitting position without chewing. Complete dissolution required approximately 10–15 minutes. After completion of topical anesthesia, patients were sequentially positioned in the supine, right lateral decubitus, and finally left lateral decubitus positions before undergoing EGD.

### Concomitant care

All participants received the same standard pre-endoscopic care, including overnight fasting for at least 6–8 hours before the procedure. No sedative medications were administered. After completion of topical anesthesia with the assigned ice popsicle, all participants underwent the same positioning protocol before EGD and were examined using the same endoscopic equipment by the same experienced endoscopist. During the examination, water flushing and additional diluted simethicone solution were permitted only after completion of the primary mucosal visibility assessment when visualization was considered insufficient to safely continue the procedure. Post-procedural care and observation were identical for both groups.

### Endoscopic procedure

All examinations were performed by a single experienced endoscopist who had performed more than 1,000 diagnostic EGDs before initiation of the study, thereby minimizing procedural variability. A second endoscopy nurse assisted during all examinations. High-definition upper gastrointestinal endoscopes (GIF-HQ190 and GIF-H290Z; Olympus, Tokyo, Japan) were used throughout the study.

Patient anxiety was assessed immediately before administration of topical anesthesia using an 11-point numerical rating scale (NRS), ranging from 0 (completely calm) to 10 (extremely anxious).

### Assessment of mucosal visibility

The primary outcome was mucosal visibility, assessed using a modified Total Visibility Score (TVS) adapted from Kuo et al [25]. The original grading system was modified to include six upper gastrointestinal segments: distal esophagus, fundus, upper gastric body, lower gastric body, antrum, and duodenum, while preserving the original four-point grading scale for each segment.

Each segment was graded as follows:

Score 1: no adherent mucus on the mucosal surface.
Score 2: a small amount of mucus without impairing visualization.
Score 3: a moderate amount of mucus requiring less than 50 mL of water for adequate visualization.
Score 4: a large amount of mucus requiring 50 mL or more of water for adequate visualization.

The total visibility score was calculated by summing the scores from all six segments, with lower scores indicating better mucosal visibility.

Mucosal visibility was assessed in real time by the blinded endoscopist immediately after visualization of each gastrointestinal segment and before any water flushing or administration of additional diluted simethicone solution. When visualization remained inadequate after completion of the primary assessment, water flushing and additional diluted simethicone solution were permitted according to routine clinical practice to facilitate completion of the examination.

### Outcome measures

The primary outcome was mucosal visibility, assessed using the modified Total Visibility Score (TVS) described above. Mucosal visibility was evaluated in six gastrointestinal segments: the distal esophagus, gastric fundus, upper gastric body, lower gastric body, gastric antrum, and duodenum. Each segment was graded on a 4-point scale (1–4), with lower scores indicating better mucosal visibility. The scores from the six segments were summed to generate the TVS, ranging from 6 to 24, with a lower total score indicating better overall mucosal visibility. Visibility was assessed by the blinded endoscopist before any water flushing or administration of additional diluted simethicone.

Secondary outcomes included procedure duration, volume of water required for mucosal cleansing, requirement for additional diluted simethicone solution, endoscopist satisfaction, ease of esophageal instrumentation, gag reflex, patient satisfaction, procedural discomfort, and pain. Procedure duration was defined as the time from insertion of the endoscope through the mouth to complete withdrawal of the endoscope at the end of the examination. The volume of water used for mucosal cleansing during EGD was recorded in milliliters, and the requirement for additional diluted simethicone was recorded as a binary outcome (yes/no).

Immediately after completion of EGD, the endoscopist and participants independently completed standardized numerical rating scale (NRS) questionnaires. Endoscopist satisfaction and ease of esophageal instrumentation were assessed using 0–10 NRSs, whereas gag reflex was assessed using a 0–4 NRS. Patient satisfaction, procedural discomfort, and pain were assessed using 0–10 NRSs. Patient-reported outcomes were completed directly by the participants, whereas procedure-related outcomes were documented by the blinded endoscopist using standardized case report forms.

Adverse events were assessed from administration of the ice popsicle until completion of post-procedural observation before discharge from the endoscopy unit. Adverse events were defined as any unfavorable clinical event occurring during this period, including allergic reactions, aspiration, respiratory symptoms, hemodynamic instability, or other symptoms requiring medical intervention. No systematic assessment of delayed adverse events after discharge was performed.

### Sample size calculation

Sample size was calculated using Stata/MP version 18.0 (StataCorp, College Station, TX, USA). The calculation was based on retrospective institutional data from patients who underwent unsedated EGD at the Gastrointestinal Endoscopy Center, Thammasat University Hospital, between June 2020 and August 2021. In the retrospective dataset, mucosal visibility was recorded as a binary clinical assessment of adequate versus inadequate visibility based on the endoscopist’s judgment, without a predefined numerical TVS threshold. Adequate mucosal visibility was observed in 59% and 44% of patients under conditions corresponding to the anticipated intervention and control groups, respectively. These retrospective proportions were used solely for sample-size estimation, whereas the TVS was the prespecified primary outcome of the prospective randomized trial. Using a two-sided chi-square test for two independent proportions with continuity correction, with a significance level (α) of 0.05 and 80% statistical power, 187 participants were required in each group, resulting in a total sample size of 374 participants.

Because all study outcomes were assessed immediately after completion of the endoscopic procedure, no loss to follow-up was anticipated; therefore, no adjustment for dropout was applied.

### Statistical analysis

All randomized participants were included in the analysis according to their originally assigned groups, following the intention-to-treat principle. Statistical analyses were performed using SPSS version 25.0 (IBM Corp., Armonk, NY, USA). Baseline demographic and clinical characteristics were summarized descriptively without hypothesis testing between randomized groups. For outcome analyses, continuous variables were assessed for normality using the Shapiro–Wilk test. Normally distributed variables are presented as mean ± standard deviation (SD) and were compared using an independent-samples t-test. Non-normally distributed or ordinal variables are presented as median with interquartile range (IQR) and were compared using the Mann–Whitney U test. Categorical variables are presented as frequencies and percentages and were compared using the chi-square test or Fisher’s exact test, as appropriate. For continuous outcomes analyzed using the t-test, treatment effects were expressed as mean differences between groups (intervention minus control) with corresponding 95% confidence intervals (CIs). For binary outcomes, treatment effects were expressed as risk ratios (RRs) with 95% CIs, where applicable.

For the prespecified primary outcome, TVS was compared between groups using an independent-samples t-test, and the treatment effect was reported as the mean difference with a 95% CI. Because TVS is a bounded score and demonstrated a marked floor effect, its distribution was additionally summarized using the median and interquartile range (IQR), and a Mann–Whitney U test was performed as a nonparametric sensitivity analysis. The primary t-test for TVS constituted the only confirmatory analysis.

All statistical tests were two-sided, and a P value of <0.05 was considered statistically significant. No secondary outcome was designated as confirmatory, and no adjustment for multiple comparisons was applied. Accordingly, all secondary outcome analyses and individual gastrointestinal segment analyses were considered exploratory, and their P values were interpreted accordingly. There were no deviations from the prespecified primary analysis. The additional nonparametric sensitivity analysis was performed to assess the robustness of the primary finding in the presence of the observed floor effect.

## Results

### Participant characteristics

A total of 378 patients were assessed for eligibility between December 2024 and June 2025. Four patients declined to provide written informed consent and were excluded. The remaining 374 participants were randomized equally into the intervention group (n = 187) and the control group (n = 187). All randomized participants received their assigned intervention, completed the study, and were analyzed according to their originally assigned groups under the intention-to-treat principle. No crossover, protocol deviations, or non-adherence to the assigned intervention occurred, and no participants were excluded from the analysis (Fig 1).

**Fig 1:**
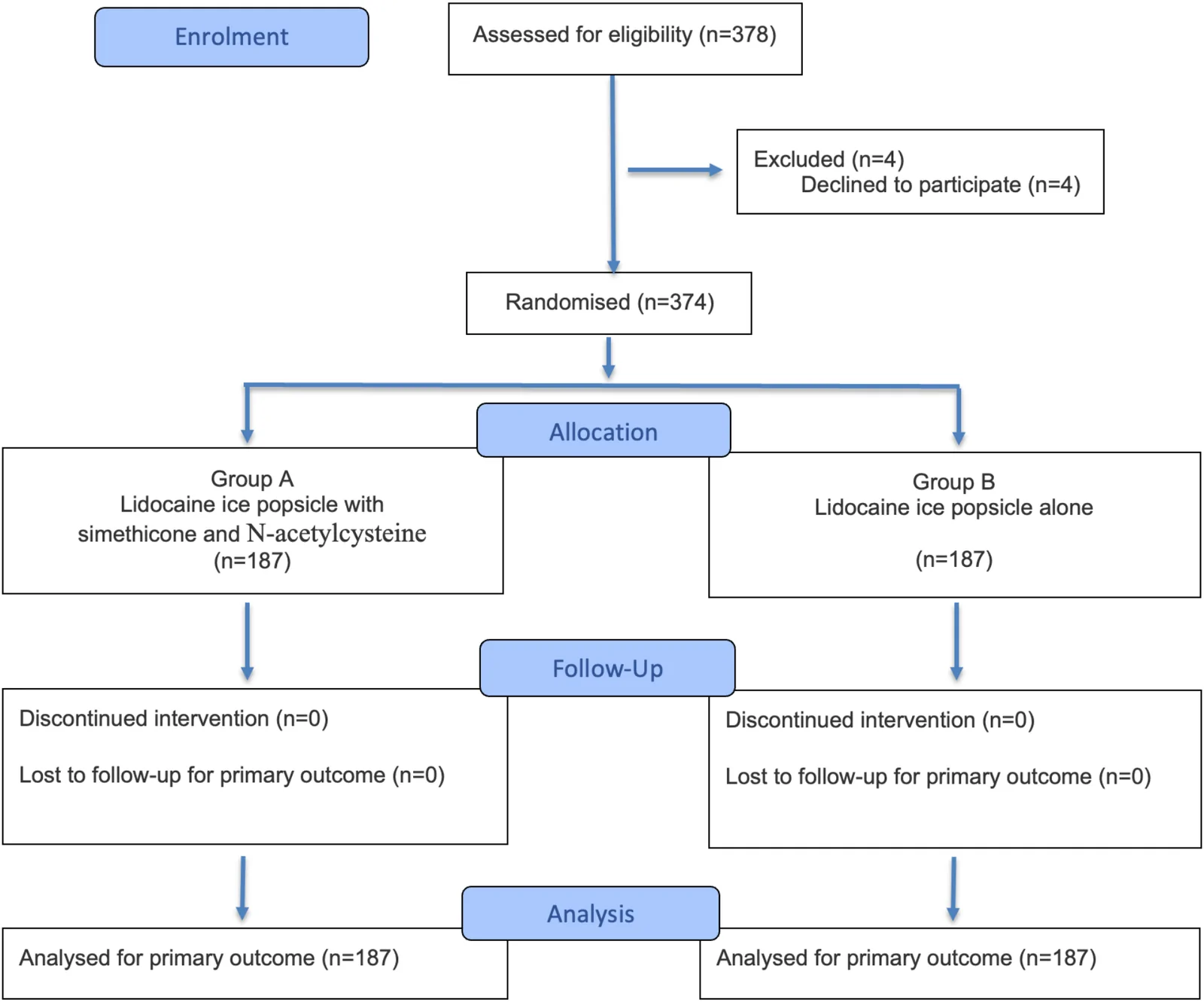
CONSORT 2025 flow diagram showing participant enrollment, randomization, follow-up, and analysis.

Among the study population, 242 participants (64.7%) were male, and the mean age was 48.5 years. The most common indication for EGD was upper gastrointestinal cancer screening (69.8%), followed by dyspepsia (15.8%) and epigastric pain (6.9%). Baseline demographic and clinical characteristics, including pre-endoscopic anxiety, are summarized descriptively in Table 1.

**Table 1:** Baseline demographic and clinical characteristics of participants by treatment group.

Unsedated EGD patients	Lidocaine + simethicone + NAC (n = 187)	Lidocaine alone (n = 187)
Age, mean ± SD, years	48.63 ± 11.28	48.12 ± 11.34
Sex Male/Female, n (%)	124 (66.3)/ 63 (33.7)	118 (63.1)/ 69 (36.9)
Body weight (kg)	61.93 ± 12.64	61.78 ± 13.51
Height (cm)	166.67 ± 9.36	164.83 ± 9.51
BMI † (kg/m2)	22.16 ± 3.49	22.57 ± 3.67
Prior experience of EGD, n (%)	139 (74.3)	123 (65.8)
Indication for EGD		
Cancer screening, n (%)	130 (69.5)	133 (71.1)
Dyspepsia, n (%)	28 (15.0)	32 (17.1)
Epigastric pain, n (%)	13 (7.0)	13 (7.0)
Reflux, n (%)	8 (4.3)	2 (1.1)
Dysphagia, n (%)	8 (4.3)	3 (1.60)
Anemia, n (%)	0 (0)	4 (2.1)
Pre-endoscopic anxiety, median [IQR]	1 (0–3.5)	1 (0–2)

†BMI Body Mass Index.

### Patient- and endoscopist-reported outcomes

Compared with the lidocaine ice popsicle alone, the lidocaine ice popsicle containing simethicone and N-acetylcysteine resulted in significantly higher endoscopist satisfaction scores (median [IQR], 10 [9–10] vs. 8 [7–8]; P < 0.001). Gag reflex scores differed significantly between groups (P = 0.022), although both groups had the same median (IQR) of 1 (1–1), suggesting that the magnitude of the difference was small. Pain scores also differed significantly between groups (P = 0.011); however, both groups had a median (IQR) pain score of 0 (0–0), indicating a minimal absolute difference. No statistically significant differences were observed in patient satisfaction, procedural discomfort, or ease of esophageal instrumentation. No adverse events were observed from administration of the study preparation through completion of post-procedural observation before discharge from the endoscopy unit.

Detailed comparisons of patient- and endoscopist-reported outcomes are summarized in Table 2.

**Table 2:** Patient- and endoscopist-reported outcomes during unsedated upper gastrointestinal endoscopy by treatment group.

Outcome	Lidocaine + simethicone + NAC, median (IQR)	Lidocaine alone, median (IQR)	P value†
Endoscopist satisfaction	10 (9–10)	8 (7–8)	<0.001
Ease of esophageal instrumentation	10 (10–10)	10 (9–10)	0.132
Gag reflex	1 (1–1)	1 (1–1)	0.022
Patient satisfaction	10 (9–10)	10 (9–10)	0.142
Procedural discomfort	1 (0–2)	1 (0–2)	0.857
Pain score	0 (0–0)	0 (0–0)	0.011

† Mann–Whitney U test.

### Mucosal visibility

The intervention group showed significantly better mucosal visibility, reflected by a significantly lower TVS. The mean TVS was 6.19 ± 0.51 in the intervention group and 10.39 ± 2.22 in the control group, corresponding to a mean difference of −4.20 (95% CI, −4.53 to −3.88; P < 0.001). The corresponding median TVS values were 6 (IQR, 6–6) and 11 (IQR, 9–13), respectively. A marked floor effect was observed in the intervention group, with 159 of 187 participants (85.0%) having the minimum possible TVS of 6. A nonparametric sensitivity analysis using the Mann–Whitney U test yielded a consistent result (U = 991.5, P < 0.001).

In exploratory segment-specific analyses, visibility scores were lower in all six gastrointestinal segments in the intervention group than in the control group (Table 3). The corresponding P values were not adjusted for multiple comparisons and should therefore be interpreted as exploratory.

**Table 3:** Total visibility score and segment-specific mucosal visibility scores during unsedated upper gastrointestinal endoscopy by treatment group.

Outcome	Visibility score, mean ± SD		Mean difference	95% CI	*p* value
	Lidocaine + simethicone + NAC (n = 187)	Lidocaine alone (n = 187)			
Distal esophagus visibility score, range 1–4 points	1.01 ± 0.07	1.48 ± 0.50	−0.47	−0.54 to −0.39	<0.001
Fundus visibility score, range 1–4 points	1.01 ± 0.10	1.45 ± 0.49	−0.44	−0.51 to −0.36	<0.001
Upper gastric body visibility score, range 1–4 points	1.01 ± 0.15	1.77 ± 0.42	−0.75	−0.82 to −0.69	<0.001
Lower gastric body visibility score, range 1–4 points	1.09 ± 0.28	2.45 ± 0.62	−1.36	−1.46 to −1.26	<0.001
Antral visibility score, range 1–4 points	1.07 ± 0.26	1.87 ± 0.54	−0.80	−0.88 to −0.71	<0.001
Duodenal visibility score, range 1–4 points	1.01 ± 0.07	1.38 ± 0.49	−0.37	−0.44 to −0.30	<0.001
Total visibility score (TVS), range 6–24 points	6.19 ± 0.51	10.39 ± 2.22	−4.20	−4.53 to −3.88	<0.001
Median (IQR)	6 (6–6)	11 (9–13)	–	–	<0.001†

†Mann–Whitney U test as a nonparametric sensitivity analysis (U = 991.5, P < 0.001).

TVS, Total Visibility Score; NAC, N-acetylcysteine; SD, standard deviation; CI, confidence interval. Lower scores indicate better mucosal visibility. Segment-specific comparisons were exploratory and were not adjusted for multiple comparisons.

Visibility was scored from 1 to 4 points for each gastrointestinal segment, with lower scores indicating better mucosal visibility. The TVS was calculated as the sum of the six segment-specific scores, yielding a possible range of 6–24 points; lower TVS values indicate better overall mucosal visibility.

### Procedural outcomes

In exploratory analyses of procedural outcomes, the intervention group demonstrated significantly improved procedural efficiency. Mean procedure time was significantly shorter than that in the control group (10.87 ± 1.44 min vs. 12.75 ± 0.96 min; mean difference, −1.87 min; 95% CI, −2.12 to −1.62; P < 0.001). The volume of water required for mucosal cleansing was also significantly lower in the intervention group (median [IQR], 50 [0–50] mL vs. 150 [100–250] mL; P < 0.001).

The requirement for additional diluted simethicone solution during EGD was significantly reduced in the intervention group (12.8% vs. 96.8%; RR, 0.13; 95% CI, 0.09–0.19; P < 0.001).

Detailed procedural outcomes are summarized in Table 4.

**Table 4:** Procedure duration, water requirement for mucosal cleansing, and use of additional simethicone during unsedated upper gastrointestinal endoscopy by treatment group.

Outcome	Lidocaine + simethicone + NAC (n = 187)	Lidocaine alone (n = 187)	MD or RR	95% CI	*p* value
Procedure duration, mean ± SD (minutes)	10.87 ± 1.44	12.75 ± 0.96	−1.87	−2.12 to −1.62	<0.001
Additional water volume, median (IQR), mL	50 (0–50)	150 (100–250)	–	–	<0.001*
Additional simethicone for mucosal cleansing, n (%)	24 (12.83)	181 (96.79)	0.13†	0.09-0.19	<0.001

* Mann–Whitney U test.

† Risk ratio.

## Discussion

In this randomized controlled trial, a lidocaine ice popsicle containing simethicone and N-acetylcysteine significantly improved mucosal visibility compared with a lidocaine ice popsicle alone during unsedated EGD. The combined formulation was also associated with shorter procedure duration, lower water requirements for mucosal cleansing, and less frequent use of additional diluted simethicone. Endoscopist satisfaction was higher with the combined formulation, whereas differences in patient-reported outcomes were generally small. No adverse events were observed during the predefined observation period.

Early detection of upper GI neoplasia remains one of the principal goals of diagnostic EGD, as early-stage lesions are associated with substantially improved treatment outcomes and long-term survival. Despite advances in high-definition endoscopy, image-enhanced endoscopy, magnifying endoscopy, chromoendoscopy, endocytoscopy, and artificial intelligence-assisted lesion detection, optimal visualization of the mucosal surface remains a prerequisite for accurate diagnosis because these technologies rely on clear visualization of the gastrointestinal mucosa [1,26–29].

Several pharmacological agents have been evaluated to improve mucosal cleanliness before EGD. Pronase-based preparations have demonstrated efficacy but are not widely available in many countries, including Thailand [9,10,12]. Simethicone effectively reduces surface tension and eliminates intraluminal bubbles, whereas NAC disrupts mucus through its mucolytic properties. Both agents have therefore been investigated as components of pre-endoscopic mucosal preparation [8,9,14]. However, these agents are typically administered as oral solutions before endoscopy. In the present study, we investigated an alternative approach by incorporating both agents into a lidocaine ice popsicle, thereby combining topical pharyngeal anesthesia and mucosal preparation in a single intervention.

Unsedated EGD remains widely practiced in many healthcare settings because it avoids sedation-related adverse events, reduces resource utilization, shortens recovery time, and lowers healthcare costs [15–17,30,31]. Effective topical anesthesia is therefore essential to improve patient tolerance and facilitate endoscope insertion. Lidocaine ice popsicles have previously been shown to provide effective pharyngeal anesthesia and may offer advantages over lidocaine spray through prolonged mucosal contact and local cooling, resulting in improved patient comfort and suppression of the gag reflex [24].

The marked improvement in mucosal visibility observed in the present study is consistent with previous reports showing that pre-endoscopic administration of simethicone, either alone or in combination with mucolytic agents such as N-acetylcysteine, improves upper gastrointestinal mucosal cleanliness and reduces bubbles and adherent mucus [8,9,14]. In our trial, the difference in the overall TVS was substantial, and exploratory segment-specific analyses showed lower visibility scores across all six gastrointestinal segments. These findings suggest that the combined formulation can improve mucosal visualization across the upper gastrointestinal segments assessed in this study. However, direct comparison with previous studies should be made cautiously because prior trials have generally used liquid oral preparations, different dosing regimens, and different visibility scoring systems. In addition, the modified six-segment TVS used in the present study has not been formally validated, which may partly account for differences in the magnitude of treatment effects across studies. The pronounced floor effect in the intervention group, with 85.0% of participants achieving the minimum possible TVS, further indicates that mucosal visibility was frequently rated at the best achievable level with the combined formulation. However, this clustering at the lower bound also limits the ability of the TVS to discriminate among participants with very good mucosal visibility. The consistency of the nonparametric sensitivity analysis with the primary analysis supports the robustness of the between-group difference despite this distributional feature.

The intervention was also associated with shorter procedure duration, lower water requirements for mucosal cleansing, and less frequent use of additional diluted simethicone. These findings are clinically plausible because improved baseline mucosal cleanliness may reduce the need for repeated flushing and suction during EGD, thereby facilitating a more efficient examination. The higher endoscopist satisfaction observed with the combined formulation is also consistent with this interpretation. In contrast, patient-reported outcomes, including satisfaction and procedural discomfort, showed little or no clinically meaningful difference between groups. This may be explained by the fact that both groups received the same dose of topical lidocaine and the same ice-popsicle delivery method, so the principal difference between groups was mucosal preparation rather than pharyngeal anesthesia. Similarly, although statistically significant differences were observed in gag reflex and pain scores in exploratory analyses, the identical or near-identical median values between groups suggest that these differences were small in magnitude and may have limited clinical relevance.

Previous studies have commonly administered simethicone and NAC in approximately 100 mL of solution to improve gastric mucosal cleanliness before EGD [14]. Such a volume is impractical for an ice-based preparation because it would substantially prolong the time required for administration. Based on preliminary feasibility testing, we selected a total volume of 30 mL, which dissolved within approximately 10–15 minutes while remaining acceptable to patients. To facilitate contact between the dissolved preparation and the upper gastrointestinal mucosa, participants were instructed to sequentially change position before endoscopy. Although the individual contribution of the positioning protocol cannot be determined from the present study, the combined strategy was associated with favorable mucosal visibility.

Several limitations should be considered when interpreting our findings. First, mucosal visibility was assessed using a modified six-segment TVS adapted from a previously published scoring system. Although the grading criteria for individual segments were unchanged, the modified scoring system has not undergone formal validation, and the findings should therefore be interpreted with appropriate caution. Additionally, although TVS was the prespecified primary outcome, the sample-size calculation was based on historical institutional data using a binary assessment of adequate mucosal visibility rather than assumptions regarding the distribution and expected treatment effect of TVS. Therefore, the design-stage statistical power for the TVS was not directly established using outcome-specific assumptions.

Second, mucosal visibility was evaluated by the performing endoscopist in real time. Although the endoscopist was blinded to treatment allocation and visibility was assessed before any water flushing or administration of additional simethicone solution, this outcome remains inherently subjective and susceptible to observer bias. Future studies incorporating independent image review and assessment of interobserver agreement would strengthen the validity of these findings.

Third, although identical ice popsicles were used and all investigators remained blinded to treatment allocation, formal assessment of blinding success was not performed. Because NAC possesses characteristic sensory properties, the possibility of partial unblinding cannot be completely excluded. Fourth, the control group received a lidocaine ice popsicle rather than standard oral simethicone solution. Differences between our findings and those of previous studies may therefore partly reflect the choice of comparator, as many previous studies compared active mucosal preparation with no premedication or conventional preparation. In the present trial, both groups received an otherwise identical lidocaine ice-popsicle preparation, with simethicone and NAC added only to the intervention formulation. Accordingly, this two-arm design estimates the effect of the combined simethicone–NAC formulation but cannot determine the separate contribution of either component. Future comparative studies including a standard oral simethicone premedication group and a lidocaine-plus-simethicone group would help clarify the incremental benefit of the individual components and the delivery method.

Finally, this was a single-center study in which all procedures were performed by one highly experienced endoscopist. Although this approach minimized procedural variability, it may limit the generalizability of the findings to other institutions and operators with different levels of experience. In addition, adverse events were assessed only from administration of the study preparation through post-procedural observation before discharge from the endoscopy unit, and delayed adverse events after discharge were not systematically assessed. Moreover, the study was not designed or powered to establish the safety of the intervention. Therefore, the absence of observed adverse events during the defined observation period should not be interpreted as evidence of safety. Multicenter randomized studies involving multiple endoscopists, standardized image assessment, externally validated visibility scoring systems, and more comprehensive safety assessment with longer follow-up are warranted.

In conclusion, the combined formulation of simethicone and N-acetylcysteine incorporated into a lidocaine ice popsicle improved mucosal visibility and procedural efficiency compared with a lidocaine ice popsicle alone during unsedated EGD. Because this two-arm trial evaluated simethicone and N-acetylcysteine only in combination, the separate contribution of each component to the observed effects cannot be determined. These findings suggest that the combined formulation may be a practical approach for pre-endoscopic preparation; however, further multicenter studies are required to confirm its effectiveness, generalizability, and safety with longer follow-up before routine clinical implementation can be recommended.

## Supporting information

S1 Dataset Anonymized minimal dataset underlying the findings of this study. (XLSX)

S1 Checklist CONSORT 2025 checklist. (DOCX)

S1 Protocol Clinical trial protocol. (DOCX)

## Abbreviations

NAC: N-acetylcysteine
RCT: randomized controlled trial
EGD: esophagogastroduodenoscopy
GI: gastrointestinal
TCTR: Thai Clinical Trials Registry
SNOSE: sequentially numbered, opaque, sealed envelope
NRS: numerical rating scale
TVS: total visibility score
