# Supplementary material for: The combination of simethicone and N-acetylcysteine in lidocaine ice popsicle compared with lidocaine ice popsicle alone in patients undergoing unsedated esophagogastroduodenoscopy: A prospective double-blind randomized controlled trial
Source: PLoS One. 2026 Oct 26;21(10):e0361495. doi: 10.1371/journal.pone.0361495 (PMC13704842; doi:10.1371/journal.pone.0361495)
Supplement: Supplementary file 2 [file pone.0361495.s003.docx]

**English Translation of the Study Protocol (Main Points)**

**Study Title**

**The Combination of Simethicone and N-Acetylcysteine in a Lidocaine Ice Popsicle Compared with a Lidocaine Ice Popsicle Alone in Patients Undergoing Unsedated Esophagogastroduodenoscopy: A Prospective Double-Blind Randomized Controlled Trial**

**Principal Investigator**

Pailin Intarasangkhanawin, MD

**Co-investigator**

Prasit Mahawongkajit, MD, PhD

Department of Surgery

Faculty of Medicine

Thammasat University

**Study Site**

Single-center study

Gastrointestinal Endoscopy Center

Thammasat University Hospital

Thailand

**Funding**

Faculty of Medicine

Thammasat University

**Background and Rationale**

Upper gastrointestinal endoscopy (EGD) is the standard diagnostic procedure for detecting diseases of the esophagus, stomach, and duodenum, including early gastrointestinal malignancies. Optimal mucosal visualization is essential for accurate diagnosis.

Although patients fast before EGD, residual mucus, bubbles, and gastric secretions often remain on the mucosal surface, reducing visibility. Simethicone acts as a defoaming agent, whereas N-acetylcysteine (NAC) dissolves mucus. Previous studies have demonstrated that combining these agents improves mucosal cleanliness before endoscopy.

Unsedated EGD remains widely practiced because it avoids sedation-related complications and reduces healthcare costs. Lidocaine ice popsicles have previously been shown to provide effective topical pharyngeal anesthesia through slow dissolution and prolonged mucosal contact.

The present study was designed to evaluate whether combining simethicone and NAC with a lidocaine ice popsicle improves mucosal visibility while maintaining patient comfort during unsedated EGD.

**Study Objective**

To compare mucosal visibility, assessed by the Total Visibility Score (TVS), between:

Lidocaine + simethicone + N-acetylcysteine ice popsicle and Lidocaine ice popsicle alone in patients undergoing unsedated EGD.

**Study Design**

- Single-center
- Prospective
- Double-blind
- Randomized controlled trial
- Allocation ratio 1:1

**Study Period**

December 2024 to June 2025

**Participants**

**Inclusion Criteria**

- Age ≥20 years
- Scheduled for unsedated diagnostic EGD
- Indications including upper GI cancer screening, dysphagia, dyspepsia, gastroesophageal reflux disease, abdominal pain, or anemia
- Able to provide written informed consent

**Exclusion Criteria**

- Previous upper gastrointestinal surgery
- Severe dysphagia
- Previous corrosive ingestion
- Allergy to lidocaine, simethicone, or NAC
- Pregnancy
- Psychiatric illness
- Neuromuscular disorders
- Unstable vital signs

**Sample Size Calculation**

Sample size was calculated using Stata/MP version 18.0.

Based on retrospective institutional data, adequate mucosal visibility was anticipated in 59% of patients receiving the combination formulation and 44% receiving lidocaine alone.

Using a two-sided alpha of 0.05 and 80% statistical power, 187 participants were required per group, resulting in a total sample size of 374 participants.

Because all study outcomes were assessed immediately after completion of the endoscopic procedure, no loss to follow-up was anticipated and no adjustment for dropout was applied.

**Randomization**

Participants were randomized in a 1:1 ratio using sequentially numbered, opaque, sealed envelopes (SNOSE).

Allocation occurred after written informed consent had been obtained.

**Blinding**

Participants, the endoscopist, assisting nurse, investigators responsible for data collection, and the statistician remained blinded to treatment allocation throughout the study.

Only the first endoscopy nurse, who prepared and administered the assigned ice popsicle, had access to the allocation sequence and was not involved in outcome assessment.

**Intervention**

**Intervention Group**

Ice popsicle containing

- Lidocaine viscous solution (120 mg)
- Simethicone 100 mg
- N-acetylcysteine 600 mg

Final volume: 30 mL

**Control Group**

Ice popsicle containing

- Lidocaine viscous solution (120 mg)

Final volume: 30 mL

**Endoscopic Procedure**

Patients sucked the assigned ice popsicle until completely dissolved (approximately 10–15 minutes).

EGD was subsequently performed using high-definition Olympus endoscopes by one experienced endoscopist.

**Primary Outcome**

Total Visibility Score (TVS)

Six upper gastrointestinal segments were evaluated using a four-point grading system.

Lower scores indicated better mucosal visibility.

**Secondary Outcomes**

- Water volume used for cleansing
- Procedure time
- Requirement for additional simethicone
- Endoscopist satisfaction
- Ease of esophageal intubation
- Gag reflex
- Patient satisfaction
- Patient discomfort
- Patient pain
- Adverse events

**Statistical Analysis**

Continuous variables were assessed for normality.

Normally distributed variables were analyzed using Student’s t-test.

Ordinal or non-normal variables were analyzed using the Mann–Whitney U test.

Categorical variables were compared using the Chi-square test or Fisher’s exact test.

A two-sided P value <0.05 was considered statistically significant.

**Withdrawal Criteria**

Participants could withdraw from the study at any time without affecting their medical care.

Investigators could withdraw participants if:

- consent was withdrawn;
- serious adverse events occurred;
- eligibility criteria were violated;
- protocol adherence was not possible; or
- continued participation was considered unsafe.

**Study Termination Criteria**

The study would be terminated if:

- unexpected safety concerns arose;
- serious adverse events related to the intervention occurred;
- the Ethics Committee required termination;
- the funding agency required termination; or
- the study could no longer be conducted according to Good Clinical Practice.

**Ethical Considerations**

The protocol was approved by the Human Research Ethics Committee, Faculty of Medicine, Thammasat University.

Written informed consent was obtained from every participant before enrollment.

The study was conducted according to the Declaration of Helsinki and Good Clinical Practice.
