# Supplementary material for: The combination of simethicone and N-acetylcysteine in lidocaine ice popsicle compared with lidocaine ice popsicle alone in patients undergoing unsedated esophagogastroduodenoscopy: A prospective double-blind randomized controlled trial
Source: PLoS One. 2026 Oct 26;21(10):e0361495. doi: 10.1371/journal.pone.0361495 (PMC13704842; doi:10.1371/journal.pone.0361495)
Supplement: Supplementary file 3 [file pone.0361495.s005.docx]

**Response to Reviewers**

Reviewer's Responses to Questions

Review Comments to the Author

Please use the space provided to explain your answers to the questions above. You may also include additional comments for the author, including concerns about dual publication, research ethics, or publication ethics. (Please upload your review as an attachment if it exceeds 20,000 characters)

**Reviewer #1:** The sample size is calculated for proportion of patients achieving adequate mucosal visibility. However, the primary endpoint is TVS. Specify the test used (chi-square with continuity?).

Response: Thank you for this important comment. We agree that the distinction between the prespecified primary outcome and the outcome used for sample-size estimation was not sufficiently clear in the original manuscript. The primary outcome of the trial was mucosal visibility assessed using the Total Visibility Score (TVS). However, because retrospective institutional data were available as proportions of patients achieving adequate mucosal visibility, these proportions were used for sample-size estimation.

The expected proportions of adequate mucosal visibility were 59% in the intervention group and 44% in the control group. Sample size was calculated using a two-sided chi-square test for two independent proportions with continuity correction, with a significance level of 0.05 and 80% power. This resulted in a required sample size of 187 participants per group, or 374 participants in total.

We have revised the Sample Size Calculation subsection to clarify the distinction between the primary outcome and the basis of the sample-size calculation and to specify the statistical test used.

Page 8 Line 218

The calculation was based on retrospective institutional data from patients who underwent unsedated EGD at the Gastrointestinal Endoscopy Center, Thammasat University Hospital, between June 2020 and August 2021. In the retrospective dataset, mucosal visibility was recorded as a binary clinical assessment of adequate versus inadequate visibility based on the endoscopist’s judgment, without a predefined numerical TVS threshold. Adequate mucosal visibility was observed in 59% and 44% of patients under conditions corresponding to the anticipated intervention and control groups, respectively. These retrospective proportions were used solely for sample-size estimation, whereas the TVS was the prespecified primary outcome of the prospective randomized trial. Using a two-sided chi-square test for two independent proportions with continuity correction, with a significance level (α) of 0.05 and 80% statistical power, 187 participants were required in each group, resulting in a total sample size of 374 participants.

Because all study outcomes were assessed immediately after completion of the endoscopic procedure, no loss to follow-up was anticipated; therefore, no adjustment for dropout was applied.

-Clarify whether adjustments for multiple comparisons were considered for secondary outcomes.

Response: Thank you for this important comment. No formal adjustment for multiple comparisons was applied to the secondary outcomes. The Total Visibility Score (TVS) was the prespecified primary outcome and constituted the primary confirmatory analysis. All secondary outcomes and segment-specific visibility analyses were considered exploratory. Accordingly, their P values were not adjusted for multiplicity and should be interpreted as exploratory rather than confirmatory evidence. We have clarified this point in the Statistical Analysis subsection of the revised manuscript.

Page 9 Line 248

For the prespecified primary outcome, TVS was compared between groups using an independent-samples t-test, and the treatment effect was reported as the mean difference with a 95% confidence interval. Because TVS is a bounded score and demonstrated a marked floor effect, its distribution was additionally summarized using the median and interquartile range (IQR), and a Mann–Whitney U test was performed as a nonparametric sensitivity analysis. The primary t-test for TVS constituted the only confirmatory analysis.

All statistical tests were two-sided, and a P value of <0.05 was considered statistically significant. No secondary outcome was designated as confirmatory, and no adjustment for multiple comparisons was applied. Accordingly, all secondary outcome analyses and individual gastrointestinal segment analyses were considered exploratory, and their P values were interpreted accordingly. There were no deviations from the prespecified primary analysis. The additional nonparametric sensitivity analysis was performed to assess the robustness of the primary finding in the presence of the observed floor effect.

**Reviewer #2:** This is a clinically relevant randomized controlled trial. However, several statistical and reporting issues should be corrected before the results can be interpreted reliably.

-The primary outcome is the Total Visibility Score (TVS), whereas the sample size calculation was based on the proportion of patients achieving “adequate mucosal visibility.” Please clarify which outcome was prespecified as primary in the protocol and registry, define adequate visibility, and ensure consistency among the protocol, sample size calculation, analysis, and reporting.

Response: Thank you for this important comment. We agree that the distinction between the prespecified primary outcome and the measure used for sample-size estimation was not sufficiently clear in the original manuscript. The Total Visibility Score (TVS) was the prespecified primary outcome of the prospective randomized trial, as specified in the original study protocol. The TVS was calculated from visibility assessments across six upper gastrointestinal segments, with lower scores indicating better mucosal visibility.

The sample-size calculation was based on retrospective institutional data available before initiation of the trial. In that retrospective dataset, mucosal visibility was recorded as a binary clinical assessment of adequate versus inadequate visibility based on the endoscopist’s judgment, without a predefined numerical TVS threshold. The proportions considered to have adequate mucosal visibility were 59% under conditions corresponding to the anticipated intervention group and 44% under conditions corresponding to the anticipated control group. These retrospective proportions were used solely for sample-size estimation and were not used to define or analyze the primary outcome of the prospective randomized trial.

Using these proportions, the sample size was calculated using a two-sided chi-square test for two independent proportions with continuity correction, with α = 0.05 and 80% statistical power, yielding 187 participants per group (374 participants in total).

We have revised the Sample Size Calculation and Outcome Measures subsections to clearly distinguish the retrospective binary clinical assessment used for sample-size estimation from the prespecified TVS primary outcome used for analysis in the randomized trial and have ensured consistent terminology throughout the manuscript.

Page 8 Line 218

The calculation was based on retrospective institutional data from patients who underwent unsedated EGD at the Gastrointestinal Endoscopy Center, Thammasat University Hospital, between June 2020 and August 2021. In the retrospective dataset, mucosal visibility was recorded as a binary clinical assessment of adequate versus inadequate visibility based on the endoscopist’s judgment, without a predefined numerical TVS threshold. Adequate mucosal visibility was observed in 59% and 44% of patients under conditions corresponding to the anticipated intervention and control groups, respectively. These retrospective proportions were used solely for sample-size estimation, whereas the TVS was the prespecified primary outcome of the prospective randomized trial. Using a two-sided chi-square test for two independent proportions with continuity correction, with a significance level (α) of 0.05 and 80% statistical power, 187 participants were required in each group, resulting in a total sample size of 374 participants.

Because all study outcomes were assessed immediately after completion of the endoscopic procedure, no loss to follow-up was anticipated; therefore, no adjustment for dropout was applied.

-Please specify the exact statistical method used for the primary outcome. TVS is a bounded score with a marked floor effect, particularly in the intervention group. Please report the treatment effect with a 95% confidence interval and provide the distribution of TVS. A nonparametric or randomization-based sensitivity analysis should be considered.

Response: Thank you for this important comment. We agree that the bounded nature of the TVS and the marked floor effect in the intervention group warrant more detailed reporting and a sensitivity analysis.

In the primary analysis, the between-group difference in TVS was evaluated using an independent-samples t-test, and the treatment effect was expressed as the mean difference with a 95% confidence interval. The intervention group had a mean TVS of 6.19 ± 0.51 compared with 10.39 ± 2.22 in the control group, corresponding to a mean difference of −4.20 (95% CI, −4.53 to −3.87; P <0.001).

We have now additionally reported the distribution of TVS using the median and interquartile range. The median TVS was 6 (IQR, 6–6) in the intervention group and 11 (IQR, 9–13) in the control group. We acknowledge the marked floor effect in the intervention group, in which 159 of 187 participants (85.0%) had the minimum possible TVS of 6.

To assess the robustness of the primary result to the distributional assumptions of the parametric analysis, we performed a prespecified-compatible nonparametric sensitivity analysis using the Mann–Whitney U test. This analysis also demonstrated a significant between-group difference (U = 991.5, P <0.001), consistent with the primary analysis.

We have revised the Statistical Analysis subsection, Results, and Table 3 accordingly to report the statistical method, treatment effect with its 95% confidence interval, distribution of TVS, and the nonparametric sensitivity analysis.

Page 9 Line 248

For the prespecified primary outcome, TVS was compared between groups using an independent-samples t-test, and the treatment effect was reported as the mean difference with a 95% confidence interval. Because TVS is a bounded score and demonstrated a marked floor effect, its distribution was additionally summarized using the median and interquartile range (IQR), and a Mann–Whitney U test was performed as a nonparametric sensitivity analysis. The primary t-test for TVS constituted the only confirmatory analysis.

All statistical tests were two-sided, and a P value of <0.05 was considered statistically significant. No secondary outcome was designated as confirmatory, and no adjustment for multiple comparisons was applied. Accordingly, all secondary outcome analyses and individual gastrointestinal segment analyses were considered exploratory, and their P values were interpreted accordingly. There were no deviations from the prespecified primary analysis. The additional nonparametric sensitivity analysis was performed to assess the robustness of the primary finding in the presence of the observed floor effect.

Page 10 Line 291

The mean TVS was 6.19 ± 0.51 in the intervention group and 10.39 ± 2.22 in the control group, corresponding to a mean difference of −4.20 (95% CI, −4.53 to −3.88; P <0.001). The corresponding median TVS values were 6 (IQR, 6–6) and 11 (IQR, 9–13), respectively. A marked floor effect was observed in the intervention group, with 159 of 187 participants (85.0%) having the minimum possible TVS of 6. A nonparametric sensitivity analysis using the Mann–Whitney U test yielded a consistent result (U = 991.5, P <0.001).

In exploratory segment-specific analyses, visibility scores were lower in all six gastrointestinal segments in the intervention group than in the control group (Table 3). The corresponding P values were not adjusted for multiple comparisons and should therefore be interpreted as exploratory.

**Table 3. Total visibility score and segment-specific mucosal visibility scores during unsedated upper gastrointestinal endoscopy by treatment group**

| Outcome | Visibility score, mean ± SD | | Mean difference | 95% CI | *p* value |
| --- | --- | --- | --- | --- | --- |
|  | Lidocaine + simethicone + NAC (n=187) | Lidocaine alone (n=187) |  |  |  |
| Distal esophagus visibility score, range 1–4 points | 1.01 ± 0.07 | 1.48 ± 0.50 | -0.47 | -0.54 to -0.39 | <0.001 |
| Fundus visibility score, range 1–4 points | 1.01 ± 0.10 | 1.45 ± 0.49 | -0.44 | -0.51 to -0.36 | <0.001 |
| Upper gastric body visibility score, range 1–4 points | 1.01 ± 0.15 | 1.77 ± 0.42 | -0.75 | -0.82 to -0.69 | <0.001 |
| Lower gastric body visibility score, range 1–4 points | 1.09 ± 0.28 | 2.45 ± 0.62 | -1.36 | -1.46 to -1.26 | <0.001 |
| Antral visibility score, range 1–4 points | 1.07 ± 0.26 | 1.87 ± 0.54 | -0.80 | -0.88 to -0.71 | <0.001 |
| Duodenal visibility score, range 1–4 points | 1.01 ± 0.07 | 1.38 ± 0.49 | -0.37 | -0.44 to -0.30 | <0.001 |
| Total visibility score (TVS), range 6–24 points | 6.19 ± 0.51 | 10.39 ± 2.22 | -4.20 | -4.53 to -3.88 | <0.001 |
| Median (IQR) | 6 (6–6) | 11 (9–13) | - | - | <0.001† |

†Mann–Whitney U test as a nonparametric sensitivity analysis (U = 991.5, P <0.001).

TVS, Total Visibility Score; NAC, N-acetylcysteine; SD, standard deviation; CI, confidence interval. Lower scores indicate better mucosal visibility. Segment-specific comparisons were exploratory and were not adjusted for multiple comparisons.

Visibility was scored from 1 to 4 points for each gastrointestinal segment, with lower scores indicating better mucosal visibility. The TVS was calculated as the sum of the six segment-specific scores, yielding a possible range of 6–24 points; lower TVS values indicate better overall mucosal visibility.

-Please recheck all numerical results in Tables 2–4. Several mean differences, confidence intervals, and p-values appear inconsistent. The tables should be regenerated from the final analysis dataset and all values verified.
The text states that visibility of the gastric antrum was comparable between groups, whereas Table 3 shows a large statistically significant difference. Please correct this inconsistency.

Response: Thank you for identifying these inconsistencies. We carefully rechecked the final participant-level dataset and regenerated Tables 2–4. All descriptive statistics, mean differences, risk ratios, 95% confidence intervals, and P values were recalculated and verified for internal consistency. We also revised the presentation of non-normally distributed variables to report median (IQR) with Mann–Whitney U test results where appropriate.

The inconsistency regarding gastric antral visibility has also been corrected. The previous statement that antral visibility was comparable between groups was erroneous. In the revised analysis, the intervention group had a significantly lower antral visibility score than the control group (1.07 ± 0.26 vs. 1.87 ± 0.54; mean difference, −0.80; 95% CI, −0.88 to −0.71; P < 0.001). Accordingly, the Results section now states that visibility scores were significantly lower in all six assessed gastrointestinal segments in the intervention group.

In addition, the total TVS was recalculated from the verified dataset and is now reported as 6.19 ± 0.51 in the intervention group and 10.39 ± 2.22 in the control group, with a mean difference of −4.20 (95% CI, −4.53 to −3.88; P < 0.001). The Abstract, Results, and Tables 2–4 have been updated accordingly to ensure consistency throughout the manuscript.

Page 10 Line 276

Patient- and endoscopist-reported outcomes

In exploratory analyses of secondary outcomes, pre-endoscopic anxiety scores were similar between the two groups. Compared with the lidocaine ice popsicle alone, the lidocaine ice popsicle containing simethicone and N-acetylcysteine resulted in significantly higher endoscopist satisfaction scores (median [IQR], 10[9-10] vs. 8[7-8]; P < 0.001). Gag reflex scores differed significantly between groups (P = 0.022), although both groups had the same median (IQR) of 1(1-1), suggesting that the magnitude of the difference was small. Pain scores also differed significantly between groups (P = 0.011); however, both groups had a median (IQR) pain score of 0(0-0), indicating a minimal absolute difference. No statistically significant differences were observed in patient satisfaction, procedural discomfort, or ease of esophageal instrumentation. No adverse events were observed from administration of the study preparation through completion of post-procedural observation before discharge from the endoscopy unit.

Detailed comparisons of patient- and endoscopist-reported outcomes are summarized in Table 2.

Page 11 Line 301

Procedural outcomes

In exploratory analyses of procedural outcomes, the intervention group demonstrated significantly improved procedural efficiency. Mean procedure time was significantly shorter than that in the control group (10.87 ± 1.44 min vs. 12.75 ± 0.96 min; mean difference, −1.87 min; 95% CI, −2.12 to −1.62; P < 0.001). The volume of water required for mucosal cleansing was also significantly lower in the intervention group (median [IQR], 50 [0–50] mL vs. 150 [100–250] mL; P < 0.001).

The requirement for additional diluted simethicone solution during EGD was significantly reduced in the intervention group (12.8% vs. 96.8%; RR, 0.13; 95% CI, 0.09–0.19; P < 0.001).

Detailed procedural outcomes are summarized in Table 4.

**Table 2. Patient- and endoscopist-reported outcomes during unsedated upper gastrointestinal endoscopy by treatment group**

| Outcome | Lidocaine + simethicone + NAC, median (IQR) | Lidocaine alone, median (IQR) | P value† |
| --- | --- | --- | --- |
| Endoscopist satisfaction | 10 (9–10) | 8 (7–8) | <0.001 |
| Ease of esophageal instrumentation | 10 (10–10) | 10 (9–10) | 0.132 |
| Gag reflex | 1 (1–1) | 1 (1–1) | 0.022 |
| Anxiety | 1 (0–3.5) | 1 (0–2) | 0.949 |
| Patient satisfaction | 10 (9–10) | 10 (9–10) | 0.142 |
| Procedural discomfort | 1 (0–2) | 1 (0–2) | 0.857 |
| Pain score | 0 (0–0) | 0 (0–0) | 0.011 |

† Mann–Whitney U test.

**Table 3. Total visibility score and segment-specific mucosal visibility scores during unsedated upper gastrointestinal endoscopy by treatment group**

| Outcome | Visibility score, mean ± SD | | Mean difference | 95% CI | *p* value |
| --- | --- | --- | --- | --- | --- |
|  | Lidocaine + simethicone + NAC (n=187) | Lidocaine alone (n=187) |  |  |  |
| Distal esophagus visibility score, range 1–4 points | 1.01 ± 0.07 | 1.48 ± 0.50 | -0.47 | -0.54 to -0.39 | <0.001 |
| Fundus visibility score, range 1–4 points | 1.01 ± 0.10 | 1.45 ± 0.49 | -0.44 | -0.51 to -0.36 | <0.001 |
| Upper gastric body visibility score, range 1–4 points | 1.01 ± 0.15 | 1.77 ± 0.42 | -0.75 | -0.82 to -0.69 | <0.001 |
| Lower gastric body visibility score, range 1–4 points | 1.09 ± 0.28 | 2.45 ± 0.62 | -1.36 | -1.46 to -1.26 | <0.001 |
| Antral visibility score, range 1–4 points | 1.07 ± 0.26 | 1.87 ± 0.54 | -0.80 | -0.88 to -0.71 | <0.001 |
| Duodenal visibility score, range 1–4 points | 1.01 ± 0.07 | 1.38 ± 0.49 | -0.37 | -0.44 to -0.30 | <0.001 |
| Total visibility score (TVS), range 6–24 points | 6.19 ± 0.51 | 10.39 ± 2.22 | -4.20 | -4.53 to -3.88 | <0.001 |
| Median (IQR) | 6 (6–6) | 11 (9–13) | - | - | <0.001† |

†Mann–Whitney U test as a nonparametric sensitivity analysis (U = 991.5, P <0.001).

TVS, Total Visibility Score; NAC, N-acetylcysteine; SD, standard deviation; CI, confidence interval. Lower scores indicate better mucosal visibility. Segment-specific comparisons were exploratory and were not adjusted for multiple comparisons.

Visibility was scored from 1 to 4 points for each gastrointestinal segment, with lower scores indicating better mucosal visibility. The TVS was calculated as the sum of the six segment-specific scores, yielding a possible range of 6–24 points; lower TVS values indicate better overall mucosal visibility.

**Table 4. Procedure duration, water requirement for mucosal cleansing, and use of additional simethicone during unsedated upper gastrointestinal endoscopy by treatment group**

| Outcome | Lidocaine + simethicone + NAC (n=187) | Lidocaine alone (n=187) | MD or RR | 95% CI | *p* value |
| --- | --- | --- | --- | --- | --- |
| Procedure duration, mean ± SD (minutes) | 10.87 ± 1.44 | 12.75 ± 0.96 | -1.87 | -2.12 to -1.62 | <0.001 |
| Additional water volume, median (IQR), mL | 50 (0–50) | 150 (100–250) | - | - | <0.001* |
| Additional simethicone for mucosal cleansing, n (%) | 24 (12.83) | 181 (96.79) | 0.13† | 0.09-0.19 | <0.001 |

* Mann–Whitney U test.

† Risk ratio.

-Please provide full details of randomization, including sequence generation, blocking or stratification, allocation concealment, and who generated the sequence, enrolled participants, and assigned interventions.

Response: Thank you for this important comment. We have expanded the description of the randomization and allocation procedures in the Methods section. The random allocation sequence was computer-generated by a research assistant who was not involved in participant enrollment or outcome assessment. Participants were randomly assigned in a 1:1 ratio to the intervention or control group. The research team screened and enrolled eligible participants after obtaining written informed consent. Allocation concealment was maintained using sequentially numbered, opaque, sealed envelopes (SNOSE), which were prepared in advance by the research assistant according to the computer-generated allocation sequence. Each envelope contained the group assignment and remained sealed until enrollment was completed. No blocking or stratification was used. After enrollment, the first endoscopy nurse opened the next sequentially numbered envelope and prepared and administered the assigned ice popsicle. This nurse was not involved in endoscopic outcome assessment or recording of the primary outcome. Participants, endoscopists, assisting nurses, data recorders, and the statistical analyst remained blinded to group allocation until completion of the final analysis.

Page 5 Line 122

The random allocation sequence was computer-generated by a research assistant who was not involved in participant enrollment or outcome assessment. The research team screened eligible participants and obtained written informed consent before enrollment. Participants were then randomly assigned in a 1:1 ratio to the intervention or control group. Allocation concealment was maintained using sequentially numbered, opaque, sealed envelopes (SNOSE), which were prepared in advance by the research assistant according to the computer-generated allocation sequence. Each envelope contained the group assignment and remained sealed until enrollment was completed. No blocking or stratification was used. After enrollment, the first endoscopy nurse opened the next sequentially numbered envelope and prepared and administered the assigned ice popsicle. This nurse was not involved in outcome assessment.

-Please state explicitly whether all randomized participants were analyzed according to their assigned groups under the intention-to-treat principle. Any protocol deviations or non-adherence should also be reported.

Response: Thank you for this comment. We have clarified the analysis population and protocol adherence in the Methods and Results sections. All 374 randomized participants were analyzed according to their originally assigned groups under the intention-to-treat principle (187 participants in each group). All participants received the allocated intervention, and no crossover, protocol deviations, or non-adherence to the assigned intervention occurred. No participants were excluded from the analysis.

Page 9 Line 234

All randomized participants were included in the analysis according to their originally assigned groups, following the intention-to-treat principle.

Page 10 Line 267

All randomized participants received their assigned intervention, completed the study, and were analyzed according to their originally assigned groups under the intention-to-treat principle. No crossover, protocol deviations, or non-adherence to the assigned intervention occurred, and no participants were excluded from the analysis (Figure 1).

-Please identify which secondary analyses were confirmatory and which were exploratory. Unless multiplicity was controlled, secondary and segment-specific p-values should be interpreted as exploratory.

Response: Thank you for this important comment. We have clarified the distinction between confirmatory and exploratory analyses in the Statistical Analysis section. The prespecified primary outcome, total visibility score (TVS), was the only confirmatory analysis. No secondary outcome was designated as confirmatory. Because no adjustment for multiple comparisons was applied, all secondary outcome analyses and individual gastrointestinal segment analyses were considered exploratory, and their P values were interpreted accordingly. The manuscript has been revised to make this distinction explicit.

Page 9 Line 252

The primary t-test for TVS constituted the only confirmatory analysis.

Page 9 Line 255

No secondary outcome was designated as confirmatory, and no adjustment for multiple comparisons was applied. Accordingly, all secondary outcome analyses and individual gastrointestinal segment analyses were considered exploratory, and their P values were interpreted accordingly.

Page 11 Line 297

In exploratory segment-specific analyses, visibility scores were lower in all six gastrointestinal segments in the intervention group than in the control group (Table 3). The corresponding P values were not adjusted for multiple comparisons and should therefore be interpreted as exploratory.

Page 10 Line 276

Patient- and endoscopist-reported outcomes

In exploratory analyses of secondary outcomes, pre-endoscopic anxiety scores were similar between the two groups.

Page 11 Line 301

Procedural outcomes

In exploratory analyses of procedural outcomes, the intervention group demonstrated significantly improved procedural efficiency.

-Please remove baseline p-values from Table 1 and the corresponding text. Baseline variables in a randomized trial should be summarized descriptively.

Response: Thank you for this important comment. We agree that baseline characteristics in a randomized trial should be summarized descriptively rather than subjected to significance testing. Accordingly, we have removed all baseline P values from Table 1 and deleted the corresponding statement regarding statistical differences between groups from the Results section. Baseline demographic and clinical characteristics are now presented descriptively only. The Statistical Analysis section has also been revised to clarify that hypothesis testing was performed for outcome analyses, whereas baseline characteristics were summarized descriptively without between-group significance testing.

Page 9 Line 236

Baseline demographic and clinical characteristics were summarized descriptively without hypothesis testing between randomized groups.

Page 10 Line 273

Baseline demographic and clinical characteristics, including pre-procedural anxiety scores, are summarized descriptively in Table 1.

**Table 1. Baseline demographic and clinical characteristics of participants by treatment group**

| Unsedated EGD patients | Lidocaine + simethicone + NAC (n=187) | Lidocaine alone (n=187) |
| --- | --- | --- |
| Age, mean ± SD, years | 48.63 ± 11.28 | 48.12 ± 11.34 |
| Sex Male/Female, n (%) | 124 (66.31) / 63 (33.69) | 118 (63.10) / 69 (36.90) |
| Body weight (kg) | 61.93 ± 12.64 | 61.78 ± 13.51 |
| Height (m) | 166.67 ± 9.36 | 164.83 ± 9.51 |
| BMI † (kg/m2) | 22.16 ± 3.49 | 22.57 ± 3.67 |
| Prior experience of EGD, n (%) | 139 (74.33) | 123 (66.76) |
| Indication for EGD |  |  |
| Cancer screening, n (%) | 131 (70.05) | 133 (71.12) |
| Reflux, n (%) | 8 (4.28) | 2 (1.07) |
| Dysphagia, n (%) | 8 (4.28) | 3 (1.60) |
| Dyspepsia, n (%) | 27 (14.44) | 32 (17.12) |
| Anemia, n (%) | 0 (0) | 4 (2.14) |

†BMI Body Mass Index

-Please moderate the safety conclusion. No adverse events were observed, but this does not establish safety. The adverse-event definition and observation period should be described.

Response: Thank you for this important comment. We agree that the absence of observed adverse events does not establish the safety of the intervention. We have therefore moderated the safety-related statements throughout the manuscript. The Methods section now defines the adverse events assessed and specifies that the observation period extended from administration of the study preparation through completion of post-procedural observation before discharge from the endoscopy unit. We have also clarified that delayed adverse events after discharge were not systematically assessed. The Results now report only that no adverse events were observed during the defined observation period. In the Discussion, we have added this limited observation period as a study limitation and clarified that the study was not designed or powered to establish safety. The Conclusion has also been revised to indicate that further studies with longer follow-up are needed to evaluate safety.

Page 8 Line 210

Adverse events were assessed from administration of the ice popsicle until completion of post-procedural observation before discharge from the endoscopy unit. Adverse events were defined as any unfavorable clinical event occurring during this period, including allergic reactions, aspiration, respiratory symptoms, hemodynamic instability, or other symptoms requiring medical intervention. No systematic assessment of delayed adverse events after discharge was performed.

Page 10 Line 286

No adverse events were observed from administration of the study preparation through completion of post-procedural observation before discharge from the endoscopy unit.

Page 14 Line 410

In addition, adverse events were assessed only from administration of the study preparation through post-procedural observation before discharge from the endoscopy unit, and delayed adverse events after discharge were not systematically assessed. Moreover, the study was not designed or powered to establish the safety of the intervention. Therefore, the absence of observed adverse events during the defined observation period should not be interpreted as evidence of safety. Multicenter randomized studies involving multiple endoscopists, standardized image assessment, externally validated visibility scoring systems, and more comprehensive safety assessment with longer follow-up are warranted.

Page 15 Line 423

These findings suggest that the combined formulation may be a practical approach for pre-endoscopic preparation; however, further multicenter studies are required to confirm its effectiveness, generalizability, and safety with longer follow-up before routine clinical implementation can be recommended.

-The conclusion should be limited to the combined formulation of simethicone and N-acetylcysteine. This two-arm trial cannot determine the separate contribution of each component.

Response: Thank you for this important comment. We agree that the two-arm design of the present trial does not allow the separate effects of simethicone and N-acetylcysteine to be determined. We have therefore revised the Conclusion to limit our interpretation specifically to the combined formulation of simethicone and N-acetylcysteine incorporated into a lidocaine ice popsicle. We have also clarified in the Discussion that the observed effects cannot be attributed to either component individually and that additional comparator groups would be required to determine their separate contributions.

Page 15 Line 418

In conclusion, the combined formulation of simethicone and N-acetylcysteine incorporated into a lidocaine ice popsicle improved mucosal visibility and procedural efficiency compared with a lidocaine ice popsicle alone during unsedated EGD. Because this two-arm trial evaluated simethicone and N-acetylcysteine only in combination, the separate contribution of each component to the observed effects cannot be determined. These findings suggest that the combined formulation may be a practical approach for pre-endoscopic preparation; however, further multicenter studies are required to confirm its effectiveness, generalizability, and safety with longer follow-up before routine clinical implementation can be recommended.

**Minor points:**
Report exact p-values except when p<0.001.
Use “±” consistently instead of “+”.
Define precisely how procedure duration was measured.
Replace “Group A” and “Group B” with informative treatment-group names.
Ensure that the Abstract, Results, tables, and conclusion contain consistent numerical values.

Response: Thank you for these helpful comments. We have addressed all of the minor points as follows. Exact P values are now reported throughout the manuscript except when P < 0.001, and the “±” symbol is used consistently for mean ± SD. The Methods section has been revised to provide a precise definition of procedure duration. In all tables, “Group A” and “Group B” have been replaced with informative treatment-group names. Finally, Tables 2–4 were regenerated and verified using the final analysis dataset, and all numerical values, including summary statistics, effect estimates, confidence intervals, and P values, were cross-checked across the Abstract, Results, tables, and Conclusion to ensure consistency.

Page 7 Line 199

Procedure duration was defined as the time from insertion of the endoscope through the mouth to complete withdrawal of the endoscope at the end of the examination.

**Abstract**

Background: Optimal mucosal visualization is essential for high-quality esophagogastroduodenoscopy (EGD), particularly for the detection of subtle upper gastrointestinal lesions. Oral simethicone and N-acetylcysteine (NAC) are commonly used to improve mucosal cleanliness, whereas lidocaine ice popsicles provide effective topical pharyngeal anesthesia for unsedated EGD. We evaluated whether incorporating simethicone and NAC into a lidocaine ice popsicle could improve mucosal visibility while maintaining patient comfort.

Methods: This single-center, prospective, double-blind, randomized controlled trial enrolled 374 adults undergoing unsedated diagnostic EGD between December 2024 and June 2025. Participants were randomized (1:1) to receive either a lidocaine ice popsicle containing simethicone (100 mg) and NAC (600 mg) or a conventional lidocaine ice popsicle. The primary outcome was mucosal visibility assessed using a modified Total Visibility Score (TVS). Secondary outcomes included procedure time, water volume required for mucosal cleansing, additional simethicone use, endoscopist satisfaction, ease of esophageal instrumentation, gag reflex, patient satisfaction, discomfort, pain, and adverse events. Statistical analyses were performed using SPSS version 25.0 (IBM Corp., Armonk, NY, USA).

Results: All 374 randomized participants (187 per group) completed the study and were analyzed according to their assigned groups. Baseline characteristics were descriptively similar between groups. The intervention group had a significantly lower TVS than the control group (6.19 ± 0.51 vs. 10.39 ± 2.22; mean difference, −4.20; 95% CI, −4.53 to −3.88; P < 0.001), indicating better mucosal visibility. Exploratory segment-specific analyses showed lower visibility scores in all six gastrointestinal segments. The intervention group also had higher endoscopist satisfaction, shorter procedure time (10.87 ± 1.44 vs. 12.75 ± 0.96 min; P < 0.001), lower water requirements for mucosal cleansing, and less frequent use of additional diluted simethicone during EGD (12.8% vs. 96.8%; P < 0.001). Exploratory analyses showed statistically significant differences in gag reflex and pain scores, although the absolute differences were small. Patient satisfaction, discomfort, and ease of esophageal instrumentation did not differ significantly between groups. No adverse events were observed during the defined observation period.

Conclusions: The combined formulation of simethicone and N-acetylcysteine incorporated into a lidocaine ice popsicle improved mucosal visibility compared with a lidocaine ice popsicle alone during unsedated EGD and was associated with improved procedural efficiency. This integrated formulation may provide a practical approach to pre-endoscopic preparation by combining topical anesthesia with mucosal cleansing. Further multicenter studies are warranted to confirm its effectiveness, generalizability, and safety with longer follow-up.

**Reviewer #3:** Dear Editor,

Thank you for selecting me as a reviewer for this manuscript. Please find below my recommendations for improving the manuscript:

1. Please specify the statistical analysis software and its version in the Methods section of the Abstract.

Response: Thank you for this helpful comment. We have revised the Methods section of the Abstract to specify the statistical software and version used for the analysis. The following sentence has been added: “Statistical analyses were performed using SPSS version 25.0 (IBM Corp., Armonk, NY, USA).”

**Abstract**

Methods: This single-center, prospective, double-blind, randomized controlled trial enrolled 374 adults undergoing unsedated diagnostic EGD between December 2024 and June 2025. Participants were randomized (1:1) to receive either a lidocaine ice popsicle containing simethicone (100 mg) and NAC (600 mg) or a conventional lidocaine ice popsicle. The primary outcome was mucosal visibility assessed using a modified Total Visibility Score (TVS). Secondary outcomes included procedure time, water volume required for mucosal cleansing, additional simethicone use, endoscopist satisfaction, ease of esophageal intubation, gag reflex, patient satisfaction, discomfort, pain, and adverse events. Statistical analyses were performed using SPSS version 25.0 (IBM Corp., Armonk, NY, USA).

2. In the “Outcome Measurements” subsection of the Methods, please provide a more detailed description of the outcomes, including the methods used for their assessment or measurement and the relevant indices or scoring systems, where applicable.

Response: Thank you for this important comment. We have expanded the “Outcome measures” subsection to provide more detailed definitions and assessment methods for both the primary and secondary outcomes. Specifically, we now describe the six gastrointestinal segments and 4-point grading system used to calculate the modified TVS, the timing of visibility assessment, the definition and measurement of procedure duration and water volume, the assessment of additional simethicone use, and the numerical rating scales used for patient- and endoscopist-reported outcomes. We have also clarified the assessment and observation period for adverse events.

Page 7 Line 187

Outcome measures

The primary outcome was mucosal visibility, assessed using the modified Total Visibility Score (TVS) described above. Mucosal visibility was evaluated in six gastrointestinal segments: the distal esophagus, gastric fundus, upper gastric body, lower gastric body, gastric antrum, and duodenum. Each segment was graded on a 4-point scale (1–4), with lower scores indicating better mucosal visibility. The scores from the six segments were summed to generate the TVS, ranging from 6 to 24, with a lower total score indicating better overall mucosal visibility. Visibility was assessed by the blinded endoscopist before any water flushing or administration of additional diluted simethicone.

Secondary outcomes included procedure duration, volume of water required for mucosal cleansing, requirement for additional diluted simethicone solution, endoscopist satisfaction, ease of esophageal instrumentation, gag reflex, patient satisfaction, procedural discomfort, and pain. Procedure duration was defined as the time from insertion of the endoscope through the mouth to complete withdrawal of the endoscope at the end of the examination. The volume of water used for mucosal cleansing during EGD was recorded in milliliters, and the requirement for additional diluted simethicone was recorded as a binary outcome (yes/no).

Immediately after completion of EGD, the endoscopist and participants independently completed standardized numerical rating scale (NRS) questionnaires. Endoscopist satisfaction and ease of esophageal instrumentation were assessed using 0–10 NRSs, whereas gag reflex was assessed using a 0–4 NRS. Patient satisfaction, procedural discomfort, and pain were assessed using 0–10 NRSs. Patient-reported outcomes were completed directly by the participants, whereas procedure-related outcomes were documented by the blinded endoscopist using standardized case report forms.

Adverse events were assessed from administration of the ice popsicle until completion of post-procedural observation before discharge from the endoscopy unit. Adverse events were defined as any unfavorable clinical event occurring during this period, including allergic reactions, aspiration, respiratory symptoms, hemodynamic instability, or other symptoms requiring medical intervention. No systematic assessment of delayed adverse events after discharge was performed.

3. More details are required in the Statistical Analysis subsection of the Methods. Please clearly specify the statistical tests used to assess the normality of the data and, separately, the statistical methods used for non-parametric data. In addition, please clarify which statistical tests were used to compare the means between the two groups and describe how the effect sizes were calculated.

Response: Thank you for this comment. We have expanded the Statistical Analysis subsection to specify the Shapiro–Wilk test for assessment of normality, the independent-samples t-test for normally distributed continuous outcomes, the Mann–Whitney U test for non-normally distributed or ordinal outcomes, and the chi-square or Fisher’s exact test for categorical outcomes, as appropriate. We have also clarified that treatment effects for continuous outcomes were expressed as mean differences with 95% confidence intervals and, where applicable, binary outcomes as risk ratios with 95% confidence intervals. In addition, the statistical approach for the prespecified primary outcome and the nonparametric sensitivity analysis has been explicitly described.

Page 8 Line 233

Statistical analysis

All randomized participants were included in the analysis according to their originally assigned groups, following the intention-to-treat principle. Statistical analyses were performed using SPSS version 25.0 (IBM Corp., Armonk, NY, USA). Baseline demographic and clinical characteristics were summarized descriptively without hypothesis testing between randomized groups. For outcome analyses, continuous variables were assessed for normality using the Shapiro–Wilk test. Normally distributed variables are presented as mean ± standard deviation (SD) and were compared using an independent-samples t-test. Non-normally distributed or ordinal variables are presented as median with interquartile range (IQR) and were compared using the Mann–Whitney U test. Categorical variables are presented as frequencies and percentages and were compared using the chi-square test or Fisher’s exact test, as appropriate. For continuous outcomes analyzed using the t-test, treatment effects were expressed as mean differences between groups (intervention minus control) with corresponding 95% confidence intervals (CIs). For binary outcomes, treatment effects were expressed as risk ratios (RRs) with 95% CIs, where applicable.

For the prespecified primary outcome, TVS was compared between groups using an independent-samples t-test, and the treatment effect was reported as the mean difference with a 95% CI. Because TVS is a bounded score and demonstrated a marked floor effect, its distribution was additionally summarized using the median and interquartile range (IQR), and a Mann–Whitney U test was performed as a nonparametric sensitivity analysis. The primary t-test for TVS constituted the only confirmatory analysis.

All statistical tests were two-sided, and a P value of <0.05 was considered statistically significant. No secondary outcome was designated as confirmatory, and no adjustment for multiple comparisons was applied. Accordingly, all secondary outcome analyses and individual gastrointestinal segment analyses were considered exploratory, and their P values were interpreted accordingly. There were no deviations from the prespecified primary analysis. The additional nonparametric sensitivity analysis was performed to assess the robustness of the primary finding in the presence of the observed floor effect.

4. The first paragraph of the Discussion should provide a brief summary of the main findings of the present study.

Response: Thank you for this suggestion. We have revised the opening paragraph of the Discussion to provide a concise summary of the main findings of the present randomized controlled trial, including the primary mucosal visibility outcome and the principal secondary procedural and patient- and endoscopist-reported outcomes.

Page 11 Line 314

In this randomized controlled trial, a lidocaine ice popsicle containing simethicone and N-acetylcysteine significantly improved mucosal visibility compared with a lidocaine ice popsicle alone during unsedated EGD. The combined formulation was also associated with shorter procedure duration, lower water requirements for mucosal cleansing, and less frequent use of additional diluted simethicone. Endoscopist satisfaction was higher with the combined formulation, whereas differences in patient-reported outcomes were generally small. No adverse events were observed during the predefined observation period.

5. The Discussion section should be further developed, with greater emphasis on the findings of the present study. The authors should provide a more thorough interpretation of their results and compare them with those of previous studies, including studies reporting consistent findings as well as those with conflicting or different results. Possible explanations for these similarities and discrepancies should also be discussed.

Response: Thank you for this important comment. We have substantially expanded the Discussion to place greater emphasis on the findings of the present study. Specifically, we now provide a more detailed interpretation of the improvement in mucosal visibility and procedural efficiency and compare these findings with previous studies of simethicone- and N-acetylcysteine-based pre-endoscopic preparations. We also discuss differences in formulation, administration volume, comparator groups, and visibility scoring systems that may account for similarities or differences across studies. In addition, we have addressed the marked floor effect observed for the primary outcome and its implications for interpretation, discussed the clinical magnitude of the exploratory patient- and endoscopist-reported outcomes, and further clarified that the two-arm design does not permit determination of the separate contributions of simethicone and N-acetylcysteine.

Page 12 Line 344

The marked improvement in mucosal visibility observed in the present study is consistent with previous reports showing that pre-endoscopic administration of simethicone, either alone or in combination with mucolytic agents such as N-acetylcysteine, improves upper gastrointestinal mucosal cleanliness and reduces bubbles and adherent mucus [8,9,14]. In our trial, the difference in the overall TVS was substantial, and exploratory segment-specific analyses showed lower visibility scores across all six gastrointestinal segments. These findings suggest that the combined formulation can improve mucosal visualization across the upper gastrointestinal segments assessed in this study. However, direct comparison with previous studies should be made cautiously because prior trials have generally used liquid oral preparations, different dosing regimens, and different visibility scoring systems. In addition, the modified six-segment TVS used in the present study has not been formally validated, which may partly account for differences in the magnitude of treatment effects across studies. The pronounced floor effect in the intervention group, with 85.0% of participants achieving the minimum possible TVS, further indicates that mucosal visibility was frequently rated at the best achievable level with the combined formulation. However, this clustering at the lower bound also limits the ability of the TVS to discriminate among participants with very good mucosal visibility. The consistency of the nonparametric sensitivity analysis with the primary analysis supports the robustness of the between-group difference despite this distributional feature.

The intervention was also associated with shorter procedure duration, lower water requirements for mucosal cleansing, and less frequent use of additional diluted simethicone. These findings are clinically plausible because improved baseline mucosal cleanliness may reduce the need for repeated flushing and suction during EGD, thereby facilitating a more efficient examination. The higher endoscopist satisfaction observed with the combined formulation is also consistent with this interpretation. In contrast, patient-reported outcomes, including satisfaction and procedural discomfort, showed little or no clinically meaningful difference between groups. This may be explained by the fact that both groups received the same dose of topical lidocaine and the same ice-popsicle delivery method, so the principal difference between groups was mucosal preparation rather than pharyngeal anesthesia. Similarly, although statistically significant differences were observed in gag reflex and pain scores in exploratory analyses, the identical or near-identical median values between groups suggest that these differences were small in magnitude and may have limited clinical relevance.

Page 14 Line 398

Differences between our findings and those of previous studies may therefore partly reflect the choice of comparator, as many previous studies compared active mucosal preparation with no premedication or conventional preparation. In the present trial, both groups received an otherwise identical lidocaine ice-popsicle preparation, with simethicone and NAC added only to the intervention formulation. Accordingly, this two-arm design estimates the effect of the combined simethicone–NAC formulation but cannot determine the separate contribution of either component. Future comparative studies including a standard oral simethicone premedication group and a lidocaine-plus-simethicone group would help clarify the incremental benefit of the individual components and the delivery method.

6. The titles of the tables should be more informative and descriptive, clearly indicating what each table presents.

Response: Thank you for this suggestion. We have revised the titles of Tables 1–4 to make them more informative and descriptive of the data presented. The revised titles now specify the participant characteristics, patient- and endoscopist-reported outcomes, total and segment-specific mucosal visibility scores, and procedural outcomes presented in each table, respectively.

Table 1. Baseline demographic and clinical characteristics of participants by treatment group

Table 2. Patient- and endoscopist-reported outcomes during unsedated upper gastrointestinal endoscopy by treatment group

Table 3. Total visibility score and segment-specific mucosal visibility scores during unsedated upper gastrointestinal endoscopy by treatment group

Table 4. Procedure duration, water requirement for mucosal cleansing, and use of additional simethicone during unsedated upper gastrointestinal endoscopy by treatment group

7. Table 3 should include the appropriate units of measurement for all relevant variables.

Response: Thank you for this comment. We have revised Table 3 to specify the measurement scale for the total and segment-specific mucosal visibility scores. The total visibility score is now reported in points with a possible range of 6–24, while each segment-specific visibility score is reported in points with a range of 1–4. We have also added a footnote clarifying that lower scores indicate better mucosal visibility.

**Table 3. Total visibility score and segment-specific mucosal visibility scores during unsedated upper gastrointestinal endoscopy by treatment group**

| Outcome | Visibility score, mean ± SD | | Mean difference | 95% CI | *p* value |
| --- | --- | --- | --- | --- | --- |
|  | Lidocaine + simethicone + NAC (n=187) | Lidocaine alone (n=187) |  |  |  |
| Distal esophagus visibility score, range 1–4 points | 1.01 ± 0.07 | 1.48 ± 0.50 | -0.47 | -0.54 to -0.39 | <0.001 |
| Fundus visibility score, range 1–4 points | 1.01 ± 0.10 | 1.45 ± 0.49 | -0.44 | -0.51 to -0.36 | <0.001 |
| Upper gastric body visibility score, range 1–4 points | 1.01 ± 0.15 | 1.77 ± 0.42 | -0.75 | -0.82 to -0.69 | <0.001 |
| Lower gastric body visibility score, range 1–4 points | 1.09 ± 0.28 | 2.45 ± 0.62 | -1.36 | -1.46 to -1.26 | <0.001 |
| Antral visibility score, range 1–4 points | 1.07 ± 0.26 | 1.87 ± 0.54 | -0.80 | -0.88 to -0.71 | <0.001 |
| Duodenal visibility score, range 1–4 points | 1.01 ± 0.07 | 1.38 ± 0.49 | -0.37 | -0.44 to -0.30 | <0.001 |
| Total visibility score (TVS), range 6–24 points | 6.19 ± 0.51 | 10.39 ± 2.22 | -4.20 | -4.53 to -3.88 | <0.001 |
| Median (IQR) | 6 (6–6) | 11 (9–13) | - | - | <0.001† |

†Mann–Whitney U test as a nonparametric sensitivity analysis (U = 991.5, P <0.001).

TVS, Total Visibility Score; NAC, N-acetylcysteine; SD, standard deviation; CI, confidence interval. Lower scores indicate better mucosal visibility. Segment-specific comparisons were exploratory and were not adjusted for multiple comparisons.

Visibility was scored from 1 to 4 points for each gastrointestinal segment, with lower scores indicating better mucosal visibility. The TVS was calculated as the sum of the six segment-specific scores, yielding a possible range of 6–24 points; lower TVS values indicate better overall mucosal visibility.
