# Supplementary material for: The combination of simethicone and N-acetylcysteine in lidocaine ice popsicle compared with lidocaine ice popsicle alone in patients undergoing unsedated esophagogastroduodenoscopy: A prospective double-blind randomized controlled trial
Source: PLoS One. 2026 Oct 26;21(10):e0361495. doi: 10.1371/journal.pone.0361495 (PMC13704842; doi:10.1371/journal.pone.0361495)
Supplement: Supplementary file 4 [file pone.0361495.s006.docx]

**Response to Reviewers**

PONE-D-26-32896R1

The combination of simethicone and N-acetylcysteine in lidocaine ice popsicle compared with lidocaine ice popsicle alone in patients undergoing unsedated esophagogastroduodenoscopy, A prospective double-blind randomized controlled trial.

Reviewers' comments:

Reviewer's Responses to Questions

**Comments to the Author**

6. Review Comments to the Author

Please use the space provided to explain your answers to the questions above. You may also include additional comments for the author, including concerns about dual publication, research ethics, or publication ethics. (Please upload your review as an attachment if it exceeds 20,000 characters)

Reviewer #1: (No Response)

**Response:** We sincerely thank the reviewer for the careful evaluation of our revised manuscript and for confirming that all previous comments have been adequately addressed.

Reviewer #2: The authors have responded carefully and respectfully to the previous comments, and the major statistical and reporting concerns have been adequately addressed. In particular, the clarification of the primary outcome, reporting of the treatment effect with a 95% confidence interval, addition of a nonparametric sensitivity analysis, distinction between confirmatory and exploratory analyses, and moderation of the safety conclusion are appropriate.

**Response:**
We sincerely thank the reviewer for the careful re-evaluation of our manuscript and for the positive assessment of the revisions. We greatly appreciate the reviewer’s constructive comments, which have helped us improve the statistical rigor, reporting clarity, and interpretation of our findings. We have carefully addressed the remaining minor comments as detailed below.

A few minor inconsistencies remain and should be corrected before acceptance:
1. Pre-endoscopic anxiety was assessed before administration of the intervention and should therefore be treated as a baseline variable. The Results state that it is presented in Table 1, but it currently appears in Table 2 with a between-group P value. Please present it descriptively in Table 1 and remove the baseline hypothesis test.

**Response:** Thank you for pointing out this inconsistency. We agree that pre-endoscopic anxiety was assessed before administration of the intervention and should therefore be treated as a baseline variable. Accordingly, we have moved pre-endoscopic anxiety from Table 2 to Table 1 and now report it descriptively without a between-group hypothesis test or P value. We have also revised the corresponding text in the Results to ensure consistency with Table 1.

Page 10 Line 273

Baseline demographic and clinical characteristics, including pre-endoscopic anxiety, are summarized descriptively in Table 1.

2. Please recheck Table 1. For prior EGD experience in the control group, 123/187 corresponds to approximately 65.8%, not 66.76%. In addition, the reported height values appear to be in centimeters, although the unit is given as meters.

**Response:** Thank you for identifying these errors. We have rechecked Table 1 and corrected the percentage of participants with prior EGD experience in the control group from 66.76% to 65.8% (123/187). We have also corrected the unit of height from meters to centimeters. Table 1 has been revised accordingly.

3. Epigastric pain is reported in the Results as an indication for EGD but is absent from Table 1. Moreover, the currently listed indications sum to only 174 participants in each group rather than 187. Please add the omitted category or categories and verify all group-specific counts and percentages against the final dataset.

**Response:** Thank you for identifying this inconsistency. We rechecked all indications for EGD against the final dataset and confirmed that epigastric pain had been inadvertently omitted from Table 1. Epigastric pain was an indication in 13 participants (7.0%) in each group. We have added this category to Table 1 and verified all group-specific counts and percentages against the final dataset. The indication categories now account for all 187 participants (100%) in each group, and the Results and Table 1 have been cross-checked for consistency.

4. The sample size was based on a historical binary assessment of adequate mucosal visibility rather than the distribution and expected treatment effect of the prespecified TVS. The authors have appropriately clarified this distinction; however, please also acknowledge as a limitation that the design-stage power for the TVS was not directly established from assumptions specific to that outcome. A post hoc power calculation is not required.

**Response:** Thank you for this important comment. We agree that, although TVS was the prespecified primary outcome, the sample-size calculation was based on historical institutional data using a binary assessment of adequate mucosal visibility rather than assumptions specific to TVS. We have therefore added this issue to the limitations of the Discussion and explicitly acknowledged that the design-stage statistical power for the TVS was not directly established using outcome-specific assumptions. As suggested by the reviewer, we did not perform a post hoc power calculation.

Page 14 Line 387

Additionally, although TVS was the prespecified primary outcome, the sample-size calculation was based on historical institutional data using a binary assessment of adequate mucosal visibility rather than assumptions regarding the distribution and expected treatment effect of TVS. Therefore, the design-stage statistical power for the TVS was not directly established using outcome-specific assumptions.

5. Please resolve the minor rounding inconsistency in the upper limit of the 95% confidence interval for the TVS mean difference (−3.87 in the response letter versus −3.88 in the manuscript and table).

After these minor corrections, I would have no further statistical concerns.

**Response:** Thank you for identifying this rounding inconsistency. We have corrected the upper limit of the 95% confidence interval in the response letter from −3.87 to −3.88 to ensure consistency with the manuscript and table. The treatment effect is now reported consistently throughout as a mean difference of −4.20 (95% CI, −4.53 to −3.88).
