# Supplementary material for: The combination of simethicone and N-acetylcysteine in lidocaine ice popsicle compared with lidocaine ice popsicle alone in patients undergoing unsedated esophagogastroduodenoscopy: A prospective double-blind randomized controlled trial
Source: PLoS One. 2026 Oct 26;21(10):e0361495. doi: 10.1371/journal.pone.0361495 (PMC13704842; doi:10.1371/journal.pone.0361495)
Supplement: Peer review history

# Peer review: Decision Letter 0

**DOI:** 10.1371/journal.pone.0361495.r001

**Authors:** Banreet Singh Dhindsa

24 Aug 2026

Dear Dr. Mahawongkajit,

Thank you for submitting your manuscript to PLOS One. After careful consideration, we feel that it has merit but does not fully meet PLOS One’s publication criteria as it currently stands. Therefore, we invite you to submit a revised version of the manuscript that addresses the points raised during the review process.

Please submit your revised manuscript by Oct 23 2026 11:59PM. If you will need more time than this to complete your revisions, please reply to this message or contact the journal office at plosone@plos.org. When you're ready to submit your revision, log on to https://www.editorialmanager.com/pone/ and select the 'Submissions Needing Revision' folder to locate your manuscript file.

- A letter that responds to each point raised by the academic editor and reviewer(s). You should upload this letter as a separate file labeled 'Response to Reviewers'.
- A marked-up copy of your manuscript that highlights changes made to the original version. You should upload this as a separate file labeled 'Revised Manuscript with Track Changes'.
- An unmarked version of your revised paper without tracked changes. You should upload this as a separate file labeled 'Manuscript'.

If you would like to make changes to your financial disclosure, please include your updated statement in your cover letter. Guidelines for resubmitting your figure files are available below the reviewer comments at the end of this letter.

If applicable, we recommend that you deposit your laboratory protocols in protocols.io to enhance the reproducibility of your results. Protocols.io assigns your protocol its own identifier (DOI) so that it can be cited independently in the future. For instructions see: https://journals.plos.org/plosone/s/submission-guidelines#loc-laboratory-protocols. Additionally, PLOS One offers an option for publishing peer-reviewed Lab Protocol articles, which describe protocols hosted on protocols.io. Read more information on sharing protocols at https://plos.org/protocols?utm_medium=editorial-email&utm_source=authorletters&utm_campaign=protocols.

As the corresponding author, your ORCID iD is verified in the submission system and will appear in the published article. PLOS supports the use of ORCID, and we encourage all coauthors to register for an ORCID iD and use it as well. Please encourage your coauthors to verify their ORCID iD within the submission system before final acceptance, as unverified ORCID iDs will not appear in the published article. *Only*  the individual author can complete the verification step; PLOS staff *cannot*  verify ORCID iDs on behalf of authors.

We look forward to receiving your revised manuscript.

Kind regards,

Banreet Singh Dhindsa

Academic Editor

PLOS One

Journal Requirements:

When submitting your revision, we need you to address these additional requirements.

1. Please ensure that your manuscript meets PLOS ONE's style requirements, including those for file naming. The PLOS ONE style templates can be found at

https://journals.plos.org/plosone/s/file?id=wjVg/PLOSOne_formatting_sample_main_body.pdf and https://journals.plos.org/plosone/s/file?id=ba62/PLOSOne_formatting_sample_title_authors_affiliations.pdf

2. Please include captions for your Supporting Information files at the end of your manuscript, and update any in-text citations to match accordingly. Please see our Supporting Information guidelines for more information: http://journals.plos.org/plosone/s/supporting-information.

3. If the reviewer comments include a recommendation to cite specific previously published works, please review and evaluate these publications to determine whether they are relevant and should be cited. There is no requirement to cite these works unless the editor has indicated otherwise.

Additional Editor Comments :

Please see reviewers comments

[Note: HTML markup is below. Please do not edit.]

Reviewers' comments:

Reviewer's Responses to Questions

**Comments to the Author**

1. Is the manuscript technically sound, and do the data support the conclusions?

Reviewer #1: Yes

Reviewer #2: Partly

Reviewer #3: Yes

**********

2. Has the statistical analysis been performed appropriately and rigorously?

Reviewer #1: Yes

Reviewer #2: No

Reviewer #3: Yes

**********

3. Have the authors made all data underlying the findings in their manuscript fully available?

The PLOS Data policy

Reviewer #1: Yes

Reviewer #2: Yes

Reviewer #3: No

**********

4. Is the manuscript presented in an intelligible fashion and written in standard English?

Reviewer #1: Yes

Reviewer #2: Yes

Reviewer #3: Yes

**********

Reviewer #1: The sample size is calculated for proportion of patients achieving adequate mucosal visibility. However, the primary endpoint is TVS. Specify the test used (chi-square with continuity?).

Clarify whether adjustments for multiple comparisons were considered for secondary outcomes.

Reviewer #2: This is a clinically relevant randomized controlled trial. However, several statistical and reporting issues should be corrected before the results can be interpreted reliably.

The primary outcome is the Total Visibility Score (TVS), whereas the sample size calculation was based on the proportion of patients achieving “adequate mucosal visibility.” Please clarify which outcome was prespecified as primary in the protocol and registry, define adequate visibility, and ensure consistency among the protocol, sample size calculation, analysis, and reporting.

Please specify the exact statistical method used for the primary outcome. TVS is a bounded score with a marked floor effect, particularly in the intervention group. Please report the treatment effect with a 95% confidence interval and provide the distribution of TVS. A nonparametric or randomization-based sensitivity analysis should be considered.

Please recheck all numerical results in Tables 2–4. Several mean differences, confidence intervals, and p-values appear inconsistent. The tables should be regenerated from the final analysis dataset and all values verified.

The text states that visibility of the gastric antrum was comparable between groups, whereas Table 3 shows a large statistically significant difference. Please correct this inconsistency.

Please provide full details of randomization, including sequence generation, blocking or stratification, allocation concealment, and who generated the sequence, enrolled participants, and assigned interventions.

Please state explicitly whether all randomized participants were analyzed according to their assigned groups under the intention-to-treat principle. Any protocol deviations or non-adherence should also be reported.

Please identify which secondary analyses were confirmatory and which were exploratory. Unless multiplicity was controlled, secondary and segment-specific p-values should be interpreted as exploratory.

Please remove baseline p-values from Table 1 and the corresponding text. Baseline variables in a randomized trial should be summarized descriptively.

Please moderate the safety conclusion. No adverse events were observed, but this does not establish safety. The adverse-event definition and observation period should be described.

The conclusion should be limited to the combined formulation of simethicone and N-acetylcysteine. This two-arm trial cannot determine the separate contribution of each component.

Minor points:

Report exact p-values except when p<0.001.

Use “±” consistently instead of “+”.

Define precisely how procedure duration was measured.

Replace “Group A” and “Group B” with informative treatment-group names.

Ensure that the Abstract, Results, tables, and conclusion contain consistent numerical values.

Reviewer #3: Dear Editor,

Thank you for selecting me as a reviewer for this manuscript. Please find below my recommendations for improving the manuscript:

1. Please specify the statistical analysis software and its version in the Methods section of the Abstract.

2. In the “Outcome Measurements” subsection of the Methods, please provide a more detailed description of the outcomes, including the methods used for their assessment or measurement and the relevant indices or scoring systems, where applicable.

3. More details are required in the Statistical Analysis subsection of the Methods. Please clearly specify the statistical tests used to assess the normality of the data and, separately, the statistical methods used for non-parametric data. In addition, please clarify which statistical tests were used to compare the means between the two groups and describe how the effect sizes were calculated.

4. The first paragraph of the Discussion should provide a brief summary of the main findings of the present study.

5. The Discussion section should be further developed, with greater emphasis on the findings of the present study. The authors should provide a more thorough interpretation of their results and compare them with those of previous studies, including studies reporting consistent findings as well as those with conflicting or different results. Possible explanations for these similarities and discrepancies should also be discussed.

6. The titles of the tables should be more informative and descriptive, clearly indicating what each table presents.

7. Table 3 should include the appropriate units of measurement for all relevant variables.

**********

what does this mean?). If published, this will include your full peer review and any attached files.

If you choose “no”, your identity will remain anonymous but your review may still be made public.

**Do you want your identity to be public for this peer review?**  For information about this choice, including consent withdrawal, please see our Privacy Policy

Reviewer #1: No

Reviewer #2: No

Reviewer #3: **Yes:**  Mohammad Mahdi Parvizi, MD, PHD, MPH

**********

[NOTE: If reviewer comments were submitted as an attachment file, they will be attached to this email and accessible via the submission site. Please log into your account, locate the manuscript record, and check for the action link "View Attachments". If this link does not appear, there are no attachment files.]

To ensure your figures meet our technical requirements, please review our figure guidelines: https://journals.plos.org/plosone/s/figures

You may also use PLOS’s free figure tool, NAAS, to help you prepare publication quality figures: https://journals.plos.org/plosone/s/figures#loc-tools-for-figure-preparation.

NAAS will assess whether your figures meet our technical requirements by comparing each figure against our figure specifications.

# Author comment: Author response to Decision Letter 1

**DOI:** 10.1371/journal.pone.0361495.r002

25 Aug 2026

Dear Editor and Reviewers,

Thank you for your careful evaluation of our manuscript and for the constructive comments and suggestions. We have revised the manuscript thoroughly in response to all editorial and reviewer comments and have provided a detailed point-by-point response.

Major revisions include clarification of the randomization, allocation concealment, blinding, intention-to-treat analysis, outcome measurements, and statistical methods; regeneration and verification of the numerical results in Tables 2–4; removal of baseline hypothesis testing; clarification of confirmatory versus exploratory analyses; additional sensitivity analysis for the primary outcome; expanded interpretation of the findings and study limitations; and moderation of the conclusions regarding safety and the combined simethicone–N-acetylcysteine formulation. We have also revised the Abstract, Results, Discussion, tables, and CONSORT reporting to ensure consistency throughout the manuscript.

All reviewer and editor comments have been addressed individually in the accompanying point-by-point response, and all changes have been incorporated into the revised manuscript.

We sincerely appreciate the Editor’s and Reviewers’ comments, which have helped us improve the clarity, methodological transparency, and overall quality of the manuscript.

Kind regards,

Prasit Mahawongkajit, M.D., Ph.D.

Attachment Submitted filename: Response to Reviewers.docx

# Peer review: Decision Letter 1

**DOI:** 10.1371/journal.pone.0361495.r003

**Authors:** Banreet Singh Dhindsa

11 Sep 2026

Dear Dr. Mahawongkajit,

Thank you for submitting your manuscript to PLOS One. After careful consideration, we feel that it has merit but does not fully meet PLOS One’s publication criteria as it currently stands. Therefore, we invite you to submit a revised version of the manuscript that addresses the points raised during the review process.

Please submit your revised manuscript by Oct 11 2026 11:59PM. If you will need more time than this to complete your revisions, please reply to this message or contact the journal office at plosone@plos.org. When you're ready to submit your revision, log on to https://www.editorialmanager.com/pone/ and select the 'Submissions Needing Revision' folder to locate your manuscript file.

- A letter that responds to each point raised by the academic editor and reviewer(s). You should upload this letter as a separate file labeled 'Response to Reviewers'.
- A marked-up copy of your manuscript that highlights changes made to the original version. You should upload this as a separate file labeled 'Revised Manuscript with Track Changes'.
- An unmarked version of your revised paper without tracked changes. You should upload this as a separate file labeled 'Manuscript'.

If you would like to make changes to your financial disclosure, please include your updated statement in your cover letter. Guidelines for resubmitting your figure files are available below the reviewer comments at the end of this letter.

If applicable, we recommend that you deposit your laboratory protocols in protocols.io to enhance the reproducibility of your results. Protocols.io assigns your protocol its own identifier (DOI) so that it can be cited independently in the future. For instructions see: https://journals.plos.org/plosone/s/submission-guidelines#loc-laboratory-protocols. Additionally, PLOS One offers an option for publishing peer-reviewed Lab Protocol articles, which describe protocols hosted on protocols.io. Read more information on sharing protocols at https://plos.org/protocols?utm_medium=editorial-email&utm_source=authorletters&utm_campaign=protocols.

As the corresponding author, your ORCID iD is verified in the submission system and will appear in the published article. PLOS supports the use of ORCID, and we encourage all coauthors to register for an ORCID iD and use it as well. Please encourage your coauthors to verify their ORCID iD within the submission system before final acceptance, as unverified ORCID iDs will not appear in the published article. *Only*  the individual author can complete the verification step; PLOS staff *cannot*  verify ORCID iDs on behalf of authors.

We look forward to receiving your revised manuscript.

Kind regards,

Banreet Singh Dhindsa

Academic Editor

PLOS One

Journal Requirements:

If the reviewer comments include a recommendation to cite specific previously published works, please review and evaluate these publications to determine whether they are relevant and should be cited. There is no requirement to cite these works unless the editor has indicated otherwise.

Please review your reference list to ensure that it is complete and correct. If you have cited papers that have been retracted, please include the rationale for doing so in the manuscript text, or remove these references and replace them with relevant current references. Any changes to the reference list should be mentioned in the rebuttal letter that accompanies your revised manuscript. If you need to cite a retracted article, indicate the article’s retracted status in the References list and also include a citation and full reference for the retraction notice.

Additional Editor Comments:

Please see reviewers comments. thank you

[Note: HTML markup is below. Please do not edit.]

Reviewers' comments:

Reviewer's Responses to Questions

**Comments to the Author**

Reviewer #1: All comments have been addressed

Reviewer #2: All comments have been addressed

**********

2. Is the manuscript technically sound, and do the data support the conclusions?

Reviewer #1: (No Response)

Reviewer #2: Yes

**********

3. Has the statistical analysis been performed appropriately and rigorously?

Reviewer #1: (No Response)

Reviewer #2: Yes

**********

4. Have the authors made all data underlying the findings in their manuscript fully available?

The PLOS Data policy

Reviewer #1: (No Response)

Reviewer #2: Yes

**********

5. Is the manuscript presented in an intelligible fashion and written in standard English?

Reviewer #1: (No Response)

Reviewer #2: Yes

**********

Reviewer #1: (No Response)

Reviewer #2: The authors have responded carefully and respectfully to the previous comments, and the major statistical and reporting concerns have been adequately addressed. In particular, the clarification of the primary outcome, reporting of the treatment effect with a 95% confidence interval, addition of a nonparametric sensitivity analysis, distinction between confirmatory and exploratory analyses, and moderation of the safety conclusion are appropriate.

A few minor inconsistencies remain and should be corrected before acceptance:

1. Pre-endoscopic anxiety was assessed before administration of the intervention and should therefore be treated as a baseline variable. The Results state that it is presented in Table 1, but it currently appears in Table 2 with a between-group P value. Please present it descriptively in Table 1 and remove the baseline hypothesis test.

2. Please recheck Table 1. For prior EGD experience in the control group, 123/187 corresponds to approximately 65.8%, not 66.76%. In addition, the reported height values appear to be in centimeters, although the unit is given as meters.

3. Epigastric pain is reported in the Results as an indication for EGD but is absent from Table 1. Moreover, the currently listed indications sum to only 174 participants in each group rather than 187. Please add the omitted category or categories and verify all group-specific counts and percentages against the final dataset.

4. The sample size was based on a historical binary assessment of adequate mucosal visibility rather than the distribution and expected treatment effect of the prespecified TVS. The authors have appropriately clarified this distinction; however, please also acknowledge as a limitation that the design-stage power for the TVS was not directly established from assumptions specific to that outcome. A post hoc power calculation is not required.

5. Please resolve the minor rounding inconsistency in the upper limit of the 95% confidence interval for the TVS mean difference (−3.87 in the response letter versus −3.88 in the manuscript and table).

After these minor corrections, I would have no further statistical concerns.

**********

what does this mean?). If published, this will include your full peer review and any attached files.

If you choose “no”, your identity will remain anonymous but your review may still be made public.

**Do you want your identity to be public for this peer review?**  For information about this choice, including consent withdrawal, please see our Privacy Policy

Reviewer #1: No

Reviewer #2: No

**********

[NOTE: If reviewer comments were submitted as an attachment file, they will be attached to this email and accessible via the submission site. Please log into your account, locate the manuscript record, and check for the action link "View Attachments". If this link does not appear, there are no attachment files.]

To ensure your figures meet our technical requirements, please review our figure guidelines: https://journals.plos.org/plosone/s/figures

You may also use PLOS’s free figure tool, NAAS, to help you prepare publication quality figures: https://journals.plos.org/plosone/s/figures#loc-tools-for-figure-preparation.

NAAS will assess whether your figures meet our technical requirements by comparing each figure against our figure specifications.

# Author comment: Author response to Decision Letter 2

**DOI:** 10.1371/journal.pone.0361495.r004

11 Sep 2026

We sincerely thank the Academic Editor and the reviewers for their careful re-evaluation of our manuscript and constructive comments. We have carefully addressed all remaining comments in the revised manuscript.

Specifically, we have reclassified pre-endoscopic anxiety as a baseline variable and removed the between-group hypothesis test; corrected and verified the baseline data in Table 1; added the previously omitted epigastric pain category and verified all indications for EGD against the final dataset; added a limitation acknowledging that the design-stage statistical power for the prespecified TVS outcome was not directly established using outcome-specific assumptions; and corrected the minor rounding inconsistency in the 95% confidence interval for the TVS mean difference.

A detailed point-by-point response to each reviewer comment is provided in the uploaded Response to Reviewers document, and all corresponding changes have been incorporated into the revised manuscript.

We greatly appreciate the Editor’s and reviewers’ comments, which have helped us improve the clarity, consistency, and statistical reporting of the manuscript.

Attachment Submitted filename: Response_to_Reviewers_auresp_2.docx

# Peer review: Decision Letter 2

**DOI:** 10.1371/journal.pone.0361495.r005

**Authors:** Banreet Singh Dhindsa

8 Oct 2026

The combination of simethicone and N-acetylcysteine in lidocaine ice popsicle compared with lidocaine ice popsicle alone in patients undergoing unsedated esophagogastroduodenoscopy, A prospective double-blind randomized controlled trial.

PONE-D-26-32896R2

Dear Dr. Mahawongkajit

We’re pleased to inform you that your manuscript has been judged scientifically suitable for publication and will be formally accepted for publication once it meets all outstanding technical requirements.

Within one week, you’ll receive an e-mail detailing the required amendments. When these have been addressed, you’ll receive a formal acceptance letter and your manuscript will be scheduled for publication.

An invoice will be generated when your article is formally accepted. Please note, if your institution has a publishing partnership with PLOS and your article meets the relevant criteria, all or part of your publication costs will be covered. Please make sure your user information is up-to-date by logging into Editorial Manager at Editorial Manager® and clicking the ‘Update My Information' link at the top of the page. For questions related to billing, please contact billing support.

If your institution or institutions have a press office, please notify them about your upcoming paper to help maximize its impact. If they’ll be preparing press materials, please inform our press team as soon as possible -- no later than 48 hours after receiving the formal acceptance. Your manuscript will remain under strict press embargo until 2 pm Eastern Time on the date of publication. For more information, please contact onepress@plos.org.

Kind regards,

Banreet Singh Dhindsa

Academic Editor

PLOS One

Additional Editor Comments (optional):

Reviewers' comments:

Reviewer's Responses to Questions

**Comments to the Author**

Reviewer #1: All comments have been addressed

**********

2. Is the manuscript technically sound, and do the data support the conclusions?

Reviewer #1: (No Response)

**********

3. Has the statistical analysis been performed appropriately and rigorously?

Reviewer #1: (No Response)

**********

4. Have the authors made all data underlying the findings in their manuscript fully available?

The PLOS Data policy

Reviewer #1: (No Response)

**********

5. Is the manuscript presented in an intelligible fashion and written in standard English?

Reviewer #1: (No Response)

**********

Reviewer #1: (No Response)

**********

what does this mean?). If published, this will include your full peer review and any attached files.

If you choose “no”, your identity will remain anonymous but your review may still be made public.

**Do you want your identity to be public for this peer review?**  For information about this choice, including consent withdrawal, please see our Privacy Policy

Reviewer #1: No

**********

# Editor report: Acceptance letter

**DOI:** 10.1371/journal.pone.0361495.r006

**Authors:** Banreet Singh Dhindsa

PONE-D-26-32896R2

PLOS One

Dear Dr. Mahawongkajit,

I'm pleased to inform you that your manuscript has been deemed suitable for publication in PLOS One. Congratulations! Your manuscript is now being handed over to our production team.

At this stage, our production department will prepare your paper for publication. This includes ensuring the following:

* All references, tables, and figures are properly cited

* All relevant supporting information is included in the manuscript submission,

* There are no issues that prevent the paper from being properly typeset

You will receive further instructions from the production team, including instructions on how to review your proof when it is ready. Please keep in mind that we are working through a large volume of accepted articles, so please give us a few days to review your paper and let you know the next and final steps.

Lastly, if your institution or institutions have a press office, please let them know about your upcoming paper now to help maximize its impact. If they'll be preparing press materials, please inform our press team within the next 48 hours. Your manuscript will remain under strict press embargo until 2 pm Eastern Time on the date of publication. For more information, please contact onepress@plos.org.

You will receive an invoice from PLOS for your publication fee after your manuscript has reached the completed accept phase. If you receive an email requesting payment before acceptance or for any other service, this may be a phishing scheme. Learn how to identify phishing emails and protect your accounts at https://explore.plos.org/phishing.

If we can help with anything else, please email us at customercare@plos.org.

Thank you for submitting your work to PLOS One and supporting open access.

Kind regards,

PLOS One Editorial Office Staff

on behalf of

Dr. Banreet Singh Dhindsa

Academic Editor

PLOS One
